# IMpact of PerioperAtive KeTamine on Enhanced Recovery After abdominal Surgery (IMPAKT ERAS): a pragmatic randomised single-cluster trial

**DOI:** 10.1016/j.bja.2025.08.001

**Published:** 2025-08-06

**Authors:** Britany L. Raymond, Brian F.S. Allen, Robert E. Freundlich, Matthew D. McEvoy, Crystal G. Parrish, Sydney R. Ruble, Kevin H. Scharfman, Jonathan P. Wanderer, Yue Gao, Leena Choi, Mary L. Dear, Hiral Master, Todd W. Rice, Miklos D. Kertai, for the Vanderbilt Learning Healthcare System Platform Investigators, Gordon Bernard, Robert Dittus, Peter Embi, Robert Freundlich, Cheryl Gatto, Frank Harrell, Paul Harris, Tina Hartert, Catherine Ivory, Ruth Kleinpell, Sunil Kripalani, Lee Ann Liska, Patrick Luther, Jay Morrison, Jill Pulley, Edward Qian, Kris Rehm, Todd Rice, Russell Rothman, Patti Runyan, Wesley Self, Matthew Semler, Jennifer Slayton, Robin Steaban, Cosby Stone, Philip Walker, Consuelo Wilkins, Adam Wright, Autumn Zuckerman, Chad Fitzgerald

**Affiliations:** 1 Department of Anesthesiology, Vanderbilt University Medical Center, Nashville, TN, USA; 2 Department of Biomedical Informatics, Vanderbilt University Medical Center, Nashville, TN, USA; 3 Paradigm Health, Franklin, TN, USA; 4 Department of Pharmaceutical Services, Vanderbilt University Medical Center, Nashville, Tennessee, United States; 5 Department of Biostatistics, Vanderbilt University Medical Center, Nashville, TN, USA; 6 Vanderbilt Institute for Clinical and Translational Research, Vanderbilt University Medical Center, Nashville, TN, USA

**Keywords:** enhanced recovery after surgery, ketamine, length of stay, multimodal analgesia, perioperative outcomes, pragmatic trial

## Abstract

**Background:**

Despite widespread adoption of ketamine into enhanced recovery after surgery (ERAS) protocols, research regarding its specific impact on perioperative outcomes is limited. This pragmatic, randomised, double-blind, placebo-controlled, single-cluster trial evaluated the impact of ketamine on postoperative outcomes in patients undergoing major abdominal surgery within an established ERAS protocol.

**Methods:**

Male and female patients, aged ≥18 yr, were randomised to ketamine or saline placebo bolus at induction of general anaesthesia, followed by an intraoperative and postoperative infusion for 48 h. The primary outcome was hospital length of stay. Secondary outcomes included total opioid consumption and the incidences of side-effects and adverse events.

**Results:**

A total of 1522 patients were included. In covariate adjusted analyses, ketamine administration did not decrease length of stay (odds ratio [OR] 1.21; 95% confidence interval [CI] 1.00–1.47) or opioid consumption (OR 0.85; 95% CI 0.71–1.01) compared with placebo. Patients receiving ketamine experienced higher odds of ICU transfer (OR 2.03; 95% CI 1.14–3.63) and lower odds of meeting early discharge milestones (OR 0.68; 95% CI 0.50–0.93). Rapid response activation (OR 1.51; 95% CI 0.85–2.68) and ileus requiring nasogastric decompression (OR 1.26; 95% CI 0.87–1.84) were similar between groups. Patients receiving ketamine experienced higher rates of debilitating dizziness (OR 6.05; 95% CI 3.02–12.11), debilitating hallucinations (OR 2.69; 95% CI 1.09–6.65), and other severe side-effects (OR 1.94; 95% CI 1.27–2.96).

**Conclusions:**

The addition of ketamine to a multimodal abdominal ERAS protocol provided no significant benefits and was associated with worse perioperative outcomes.

**Clinical trial registration:**

NCT04625283.

**Editor’s key points**

• Ketamine has been proven to reduce opioid consumption after major noncardiac surgery. However it has significant side-effects that might limit its acceptability as part of enhanced recovery from surgery protocols in abdominal surgery patients.
• In this study adult patients having abdominal surgery were randomised to ketamine or saline placebo as an intraoperative bolus and infusion, followed by a 48-h postoperative infusion. Patients in both groups received multimodal, low-opioid protocolised care.
• The addition of ketamine did not significantly reduce hospital length of stay, total opioid consumption or ileus requiring gastric decompression, while ketamine increased transfer to ICU, adverse effects, and failure to meet milestones for early discharge.
• This trial suggests that ketamine should not be included in enhanced recovery from surgery protocols for major abdominal surgery patients.

Enhanced recovery after surgery (ERAS) protocols designed to improve surgical outcomes have been widely adopted as a standardised approach to perioperative care. Although multimodal analgesia has been proven effective in improving recovery in ERAS protocols, the optimal regimen remains an area of active investigation, particularly regarding how individual agents contribute to the overall success of the strategy.^1^

While ketamine’s effectiveness in reducing opioid consumption is well documented, its role within a comprehensive multimodal analgesic regimen remains largely underexplored, despite its extensive and widespread use in ERAS protocols.^2^ Given that current ERAS protocols significantly reduce opioid requirements, the additional potential of ketamine to further reduce opioid use may be offset by its potential adverse effects. Adverse reactions, including nausea, sedation, dizziness, and delirium, could delay recovery and limit its clinical utility.^3^^,^^4^ Furthermore, ketamine’s impact on key postoperative outcomes, such as length of stay (LOS), return of bowel function, and functional recovery, has not been clearly established.

This pragmatic, randomised, double-blind, placebo-controlled, single-cluster trial aimed to address this gap by evaluating the impact of ketamine on postoperative outcomes in patients undergoing major abdominal surgery within an established ERAS protocol.^5^ We hypothesised that patients who received ketamine as part of their perioperative analgesic plan would experience a significant reduction in hospital LOS. We also hypothesised that ketamine administration would be well tolerated without adverse postoperative events, encouraging recovery by reducing total opioid requirements, and subsequently the incidence of postoperative ileus.

## Methods

### Trial approval

The IMpact of PerioperAtive KeTamine on Enhanced Recovery after Abdominal Surgery (IMPAKT ERAS) Trial protocol was approved by the Institutional Review Board (#200210, October 18, 2020) at Vanderbilt University Medical Center, Nashville, Tennessee, USA, and published before enrolment concluded.^5^ This interventional trial was registered with www.clinicaltrials.gov on November 6, 2020 (NCT04625283).

### Study design

**Fig 1:**
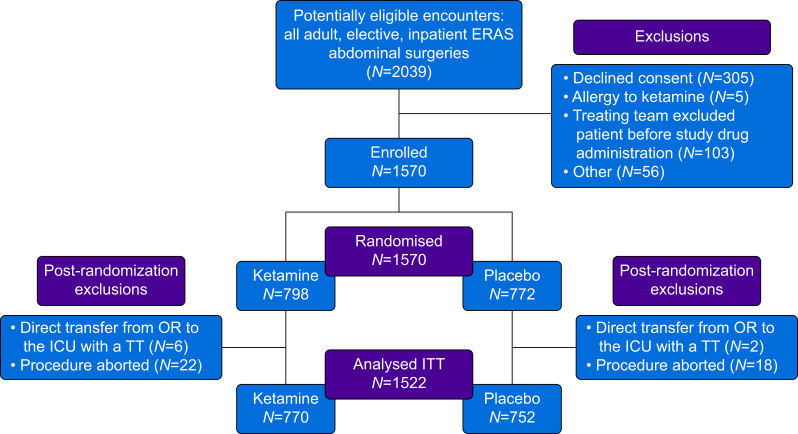
The CONSORT flow diagram of the study cohort. ERAS, Enhanced Recovery After Surgery; TT, tracheal tube; ICU, intensive care unit; ITT, intention to treat; OR, operating room.

The IMPAKT ERAS Trial was a pragmatic, randomised, double-blind, placebo-controlled, single-cluster study. Patients undergoing major abdominal surgery within an ERAS pathway were randomised on a weekly basis within 2-week or 4-week blocks to receive either ketamine or saline placebo as an intraoperative bolus and infusion, followed by a 48-h postoperative infusion (Fig. 1). Pragmatic features of the trial are highlighted in Supplementary Table S1 and Supplementary Figure S1, with the randomisation scheme displayed in Supplementary Figure S2. Study personnel and anaesthesia providers were blinded to the treatment assignments until the trial’s conclusion. Apart from the intervention, all other ERAS pathway components were identical between groups. The primary outcome was hospital LOS, with secondary outcomes assessing ketamine’s analgesic efficacy, side-effect profile, and safety.

### Eligibility

Male and female patients, aged ≥18 yr and undergoing elective abdominal surgery within colorectal, surgical oncology, or complex ventral hernia ERAS pathways were eligible. Patients unable or unwilling to receive preoperative regional analgesia were excluded, as this deviated from usual ERAS care. Post-randomisation exclusions included cancelled surgeries with same-day discharge and direct ICU admission of intubated patients from the operating room. Blinded anaesthesia providers could exclude patients before surgery if ketamine or placebo was deemed medically inappropriate, such as in patients with a history of chronic pain or severe opioid-related nausea. Once the intervention was administered, patients were enrolled and included in the analysis unless they met post-randomisation exclusion criteria.

### Randomisation

Treatment arms were assigned on a weekly basis with a 1:1 randomisation of ketamine *vs* placebo, using a random sequence generated by the study statistician. The randomisation used a block randomisation scheme with 2- and 4-week blocks (Supplementary Fig. S2). The operating room pharmacy prepared weekly batches of the designated study drug (ketamine or saline placebo) as determined by the unblinded study pharmacist. These medications were stored in perioperative Omnicell XT Automated Dispensing Cabinets (Mountain View, CA, USA) and replenished weekly. To maintain blinding of both patients and providers, study drugs were labelled with generic, non-identifying labels.

### Perioperative management

On the day of surgery, a perioperative medicine service physician conducted preoperative consultations, screened for trial eligibility, and obtained written informed consent. Enrolled patients followed usual abdominal ERAS care, including preoperative paracetamol, gabapentin, and regional nerve blocks (neuraxial for open and truncal for laparoscopic procedures).^5^

During induction of general anaesthesia, a 0.5 mg kg^−1^ i.v. bolus of the study drug (maximum 100 kg body weight) was administered, followed by a 5 μg kg^−1^ min^−1^ infusion (maximum 100 kg body weight) until fascial closure, using the same medication bag. Intraoperative care adhered to ERAS protocols, incorporating lidocaine infusions, ketorolac i.v. bolus, methadone i.v. bolus once as needed, with additional opioid administration guided per the ERAS algorithm (for details, see Supplementary Appendices S1–S3).

After surgery, ketamine or placebo infusion resumed at 2.5 μg^−1^ kg^−1^ min^−1^ (maximum 100 kg body weight) in the PACU and continued for 48 h. Patients received daily evaluations by a blinded perioperative medicine service team, managing analgesia, antiemetics, and compliance with ERAS protocols. Standard multimodal, opioid-sparing pain management included scheduled paracetamol, gabapentin, ketorolac, and a 24-h lidocaine infusion, with opioids for breakthrough pain as needed (for details, see Supplementary Appendices S1–S3).

Providers could stop the study intervention if clinically necessary, documenting the rationale (e.g. adverse effects or preparing for discharge) in the electronic health record (Epic, eStar, Verona, WI, USA) when discontinuing the study drug order earlier than its intended 48-h course.

### Outcomes

The primary outcome was hospital LOS, defined as the time from anaesthesia start to hospital discharge, a standard measure of functional recovery and ERAS efficacy.^6^ The study had several secondary outcomes for efficacy and safety. The analgesic efficacy outcome was defined by oral morphine milligram equivalent^7^ (MME, opioid conversion factors; Supplementary Table S2) consumption during the hospital LOS, with reduced MME use aligning with ERAS goals of minimising opioid-related adverse effects such as nausea and ileus. The process efficacy outcome was defined by achieving early discharge (<48 h postoperative) milestones. Safety outcomes included the incidence of adverse events (i.e. rapid response team activation, ICU transfer, ileus requiring a nasogastric tube placement) and side-effects (i.e. early discontinuation because of mild adverse effects, debilitating hallucinations, debilitating dizziness, haemodynamic instability, and other severe side-effects) as previously defined.^5^ Exploratory outcomes included the incidence of reoperation, hospital readmission within 30 days post discharge, and death. Study outcomes were derived through retrospective query of the electronic health record databases using structured query language**.**

### Per-protocol cohort

To better understand the potential efficacy of ketamine, we defined a per-protocol cohort for analysis that included patients who did not have the study infusion stopped for adverse effects. The per-protocol cohort included participants who finished the 48-h postoperative infusion, and participants for whom the study infusion was discontinued early because the patient met early recovery milestones and was preparing for discharge.

### Statistical analysis

Descriptive statistics were used to summarise the study population, including patient and clinical characteristics. Categorical variables were described using frequencies and proportions, while continuous variables were described using means and standard deviations, and medians and interquartile ranges (IQR). Missingness was reported, and single imputation was used for missing covariates in regression analyses, considering very low missingness (i.e. <1% of missingness).

Ordinal regressions with robust standard errors were used to compare the primary outcome and total opioid consumption (oral MMEs) between the ketamine and placebo arms, adjusting for covariates such as age, BMI, comorbidities, smoking status, nerve block type, intraoperative time, ASA physical status classification, preoperative opioid use, and histories of anxiety, depression, or posttraumatic stress disorder (PTSD). The odds ratio (OR) estimated from an ordinal regression analysis represents the odds of being in a higher category of the ordinal outcome (e.g. longer hospital LOS or greater total opioid consumption) *vs* a lower category for patients in the intervention arm (i.e. ketamine arm) compared with those in the control arm (i.e. placebo arm). An OR >1 indicates that the patients in the intervention arm are more likely to fall into a higher category of the outcome compared with those in the control arm.

Logistic regression with robust standard errors analysed binary secondary outcomes, adjusting for age, comorbidities, nerve block type, and preoperative opioid use when event counts allowed. Because of low event rates, no covariates were included in analyses of debilitating hallucinations or haemodynamic instability. The exploratory outcomes of reoperation and death were summarised using descriptive statistics by arm.

Differential treatment effects were explored by examining the interaction between the treatment arm indicator and each variable that potentially interacts with treatment, one variable at a time. The variables considered included sex, preoperative opioid status, surgery type, intended nerve block type, known history of depression, and known history of PTSD. Subgroup analysis was performed for variables with evidence supporting a differential treatment effect.

*Post hoc* analyses were performed for the following outcomes: resource length of stay (RLOS), case mix index-adjusted relative length of stay (CARLOS), and incidence of readmission within 30 days of discharge. RLOS and CARLOS were analysed using ordinal regressions with the same covariates as the primary analysis, while readmission within 30 days of discharge was analysed using logistic regression with age, total comorbidities, intended nerve block, and preoperative opioid status as covariates.

In addition, a per-protocol analysis was performed for the primary and selected secondary outcomes (i.e. total consumption of opioids in oral MMEs, rapid response team activation, ICU transfer, and ileus requiring gastric decompression).

The sample size calculation assumed 85% power and a two-sided type I error rate of 5%. To account for potential temporal correlation within the single cluster, we estimated an intraclass correlation coefficient of 0.014 from preliminary LOS data for major abdominal surgeries. For this analysis, subunits within the single cluster were defined by half-monthly or monthly periods. Given these assumptions, about 757 patients would need to be included in each arm to detect a 10% reduction in hospital LOS. A 2% increase in sample size was deemed necessary to accommodate two prespecified post-randomisation exclusions. Accordingly, the trial targeted 1544 participants for enrolment.

Statistical inference from all regression analyses, aimed at comparing the differences in outcomes between the ketamine and placebo arms, was based on robust standard errors accounting for temporal subunit effects within the single cluster, with the subunit variable being the week. All statistical analyses were performed using R programming language (version 4.4.1, R Core Team, http://www.r-project.org).^8^ The reporting of study and statistical results adhered to the guidelines provided in the SPIRIT (Standard Protocol Items: Recommendation for Interventional Trials) and CONSORT (Consolidated Standards of Reporting Trials) statements.^9^^,^^10^ Adjustments for multiple comparisons were not made, as all analyses—except for a few *post hoc* analyses described above—were based on scientific rationale and prespecified in the statistical analysis plan before the final data lock.

## Results

### Study population: baseline and clinical characteristics

Between April 12, 2021 and January 26, 2024, 2039 patients were assessed for eligibility, of whom 1570 were randomised to receive either blinded ketamine or saline placebo. Pre- (*n*=469) and post-randomisation (*n*=48) exclusions are reported in Figure 1. Demographic and clinical characteristics of patients in both groups are shown in Table 1. The median age of the study population was 58.3 yr (IQR 45.3–68.7 yr), and 50.7% (*n*=771) of the study population were male. The most common surgical category of the cohort was colorectal with 57.8% (*n*=879) of the study population, followed by 41.1% (*n*=626) undergoing surgical oncology procedures, and 1.1% (*n*=17) receiving ventral hernia repairs.

**Table 1:** Patient characteristics. ASA, American Society of Anesthesiologists; BMI, body mass index; PTSD, posttraumatic stress disorder; Q, quartile; sd, standard deviation. ∗Utilising opioids recreationally or medically directed with an active prescription at the time of admission to the hospital.

	Ketamine (*N*=770)	Placebo (*N*=752)
Age (yr)		
Median (Q1, Q3)	58.9 (46.7, 69.2)	57.8 (44.3, 67.8)
Sex at birth, *n* (%)		
Female	371 (48.2)	380 (50.5)
Male	399 (51.8)	372 (49.5)
Race and ethnicity, *n* (%)		
White	686 (89.1)	660 (87.8)
Black or African American	58 (7.5)	67 (8.9)
Asian	3 (0.4)	11 (1.5)
Other	15 (1.9)	9 (1.2)
Hispanic/Latino	3 (0.4)	3 (0.4)
Unknown	5 (0.6)	2 (0.3)
Weight (kg)		
Median (Q1, Q3)	81.7 (68.0, 96.9)	80.7 (68.0, 95.7)
Height (m)		
Median (Q1, Q3)	1.70 (1.63, 1.80)	1.70 (1.63, 1.80)
BMI (kg m^−2^)		
Median (Q1, Q3)	27.3 (23.7, 31.3)	27.6 (23.6, 32.0)
Total comorbidities		
Median (Q1, Q3)	1.00 (0, 2.00)	1.00 (0, 2.00)
Smoking status, *n* (%)		
History of smoking	374 (48.6)	319 (42.4)
Never smoking	394 (51.2)	433 (57.6)
Missing	2 (0.3)	0 (0)
Surgery type, *n* (%)		
Colorectal	437 (56.8)	442 (58.8)
Hernia	9 (1.2)	8 (1.1)
Surgical oncology	324 (42.1)	302 (40.2)
Intended block type, *n* (%)		
Epidural	267 (34.7)	254 (33.8)
Truncal	503 (65.3)	498 (66.2)
Intraoperative time (h)		
Median (Q1, Q3)	5.01 (3.70, 7.16)	5.03 (3.82, 6.75)
Last ASA physical status, *n* (%)		
1	2 (0.3)	2 (0.3)
2	198 (25.7)	209 (27.8)
3	546 (70.9)	522 (69.4)
4	24 (3.1)	19 (2.5)
Opioid status, *n* (%)		
Exposed∗	82 (10.6)	76 (10.1)
Naïve	688 (89.4)	676 (89.9)
Known history of anxiety, *n* (%)		
Yes	291 (37.8)	295 (39.2)
No	479 (62.2)	457 (60.8)
Known history of depression, *n* (%)		
Yes	351 (45.6)	340 (45.2)
No	419 (54.4)	412 (54.8)
Known history of PTSD, *n* (%)		
Yes	29 (3.8)	38 (5.1)
No	741 (96.2)	714 (94.9)

### Primary outcome: length of stay

**Fig 2:**
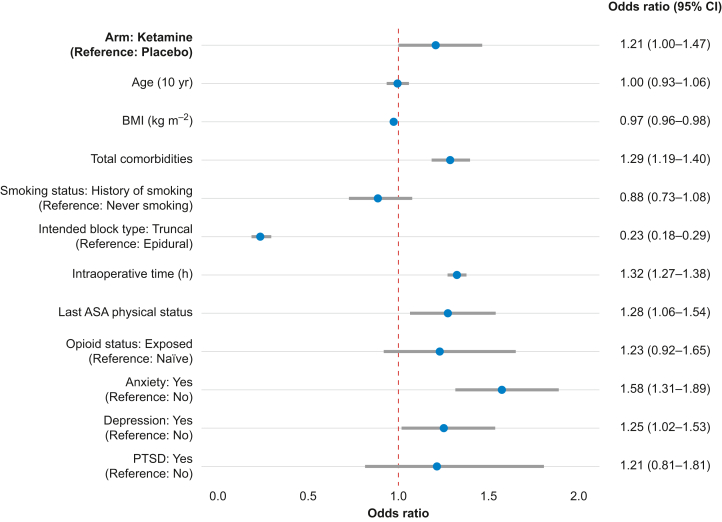
Forest plot of the regression analysis for the primary outcome, hospital length of stay, comparing the ketamine and placebo arms with adjustment of covariates. The odds ratio (OR) with the 95% confidence interval (CI) for the main effect is displayed at the top, followed by the ORs for all other adjusted covariates. ASA, American Society of Anesthesiologists; PTSD, posttraumatic stress disorder.

The median duration of hospital LOS was similar between arms, at 5 days (IQR, 4–8 days) and 5 days (IQR 3–7 days) for ketamine and placebo arms, respectively. In the regression analysis, patients randomised to receive ketamine had an OR of 1.21 (95% CI 1.00–1.47) for prolonged hospital LOS compared with those who received a placebo (Fig. 2).

After adjusting for other variables in the regression model, higher BMI and the administration of a truncal regional block (*vs* epidural) were associated with lower odds of a prolonged hospital LOS. In contrast, higher ASA physical status classification, prolonged intraoperative time, higher number of total comorbidities, history of anxiety, and history of depression were associated with higher odds of a prolonged hospital LOS (Fig. 2).

### Secondary outcomes

The median opioid consumptions were 82.5 MMEs (IQR, 37.5–158 MMEs) and 90.0 MMEs (IQR, 45.0–158 MMEs) for ketamine and placebo arms, respectively. After adjusting for covariates in the regression analysis, ketamine administration demonstrated an OR of 0.85 (95% CI 0.71–1.01) for opioid consumption compared with placebo. Other covariates in the model, such as older age, higher ASA physical status classification, higher number of total comorbidities, prolonged intraoperative time, history of smoking, anxiety, or preoperative opioid use were independently associated with higher odds of consuming more total opioids in MMEs (Supplementary Table S3).

Patients randomised to ketamine had a higher odds of experiencing a rapid response event compared with patients randomised to placebo (OR 1.51, 95% CI 0.85–2.68) (Table 2). Younger age and higher number of total comorbidities were independently associated with a higher odds of rapid response team activation (Supplementary Table S4).

**Table 2:** Incidence of adverse events and rates of early infusion discontinuation, stratified by clinical rationale. CI, confidence interval; OR, odds ratio. ∗Patients experiencing mild and tolerable side-effects were counselled on the intended purpose and possible benefits of ketamine as part of their analgesic regimen. Patients were given the option to continue or cease the medication based upon their preference.

Secondary outcomes	Ketamine *n* (%)	Placebo *n* (%)	OR (95% CI)
Adverse events:			
Rapid response activation	39 (5.1)	26 (3.5)	1.51 (0.85–2.68)
Transfer to the ICU	44 (5.7)	22 (2.9)	2.03 (1.14–3.63)
Ileus requiring nasogastric decompression	63 (8.2)	48 (6.4)	1.26 (0.87–1.84)
Early discontinuation:			
Met early discharge milestones	96 (12.5)	130 (17.3)	0.68 (0.50–0.93)
Patient preference for mild side-effects∗	83 (10.8)	39 (5.2)	2.20 (1.42–3.39)
Debilitating hallucinations	19 (2.5)	7 (0.9)	2.69 (1.09–6.65)
Debilitating dizziness	64 (8.3)	11 (1.5)	6.05 (3.02–12.11)
Haemodynamic instability	12 (1.6)	6 (0.8)	1.97 (0.79–4.93)
Other severe side-effect	71 (9.2)	37 (4.9)	1.94 (1.27–2.96)

Randomisation to ketamine was also associated with higher odds (OR 2.03; 95% CI 1.14–3.63) of experiencing ICU transfer compared with placebo (Table 2). Higher number of total comorbidities and history of preoperative opioid use were independently associated with higher odds of transfer to an ICU. Older age and administration of a truncal nerve block (*vs* epidural) were associated with lower odds of ICU transfer (Supplementary Table S4).

More patients randomised to ketamine received antiemetics because of postoperative nausea and vomiting (PONV) within the first 48 h after surgery compared with those randomised to placebo (54.8% *vs* 46.7%; *P*=0.002). However, randomisation to ketamine was associated with similar odds of ileus requiring gastric decompression (OR 1.26; 95% CI 0.87–1.84) compared with placebo (Table 2). Higher number of total comorbidities was the only covariate that was independently associated with development of ileus requiring gastric decompression (Supplementary Table S4).

Patients randomised to receive ketamine had lower odds of meeting early milestones for discharge (OR 0.68; 95% CI 0.50–0.93) (Table 2). Administration of a truncal nerve block (*vs* epidural) was independently associated with a higher odds of meeting milestones for discharge (Supplementary Table 5).

The study drug was discontinued early for adverse effects in 32.3% (*n*=249) of patients randomised to receive ketamine and in 13.3% (*n*=100) of patients randomised to placebo. Administration of ketamine was associated with a higher odds of discontinuation because of mild adverse effects (OR 2.20; 95% CI 1.42–3.39), debilitating dizziness (OR 6.05; 95% CI 3.02–12.11), debilitating hallucinations (OR 2.69; 95% CI 1.09–6.65), and other severe side-effects (OR 1.94; 95% CI 1.27–2.96) (Table 2).

### Differential treatment effects

Differential treatment effects were explored by examining the interaction between ketamine administration and surgery type (colorectal, surgical oncology, ventral hernia), intended nerve block type (truncal *vs* epidural), sex defined at birth, history of preoperative opioid use, history of depression, and history of PTSD with respect to hospital LOS (Supplementary Table S6). The analyses indicate that among opioid-naïve patients, ketamine had higher odds of a prolonged LOS (OR 1.28; 95% CI 1.05–1.56) compared with placebo. Conversely, for patients with a history of preoperative opioid use, the odds of a prolonged LOS with ketamine were 0.66 (95% CI 0.36–1.21) compared with placebo. Differential treatment effects were not found among surgery type, intended type of nerve block, sex, history of depression, or history of PTSD (Supplementary Table S6).

### Per-protocol analysis

With the high rates of early discontinuation as noted above, we investigated whether the primary and secondary outcomes differed for patients who received the study drug as intended. After excluding patients who discontinued the intervention early because of adverse effects, the odds of prolonged hospital LOS (OR 1.29; 95% CI 1.03–1.61) and ICU transfer (OR 1.96; 95% CI 1.03–3.72) for patients randomised to ketamine remained. Ketamine administration was not associated with reduced postoperative opioid consumption in MMEs (OR 0.87; 95% CI 0.70–1.07), rapid response team calls (OR 1.40; 95% CI 0.71–2.75) or ileus requiring gastric decompression (OR 1.29; 95% CI 0.83–2.00) (Supplementary Table S7) in the per-protocol analyses.

### *Post hoc* analyses

The results of covariate adjusted analyses revealed that ketamine administration was not independently associated with RLOS (OR 1.18; 95% CI 0.96–1.45) or CARLOS (OR 1.04; 95% CI 0.86–1.26). Similarly, ketamine administration was not independently associated with readmission within 30 days of discharge (OR 1.18; 95% CI 0.87–1.59) (Supplementary Table S8).

## Discussion

In this single-centre, randomised pragmatic trial, the addition of perioperative ketamine to a major abdominal ERAS protocol did not significantly reduce LOS, total opioid consumption or ileus requiring gastric decompression. Conversely, patients randomised to ketamine administration were more frequently transferred to the ICU, experienced mild to severe adverse effects, and were less likely to meet milestones for early discharge.

While the analgesic benefits of ketamine are well established,^2^^,^^11^^,^^12^ data regarding its impact on in-hospital perioperative outcomes and surgical recovery remain limited, despite these being central goals of ERAS programmes focused on accelerating discharge and improving recovery. Our trial’s emphasis on in-hospital perioperative outcomes and surgical recovery represents a key strength, offering novel insights that extend beyond ketamine’s analgesic effects.

Furthermore, previous studies have largely explored ketamine as part of opioid-based regimens, outside ERAS protocols.^2^^,^^13^ Our results align with limited research on ketamine’s role specifically within ERAS protocols and on perioperative outcomes. A retrospective chart review of 366 patients in an ERAS bariatric program found that ketamine modestly reduced LOS and opioid use, though the differences were deemed clinically irrelevant.^14^ Similarly, a cohort study of 1447 colorectal ERAS patients reported no significant LOS reduction with ketamine, and 24% experienced adverse effects leading to early discontinuation.^4^ A small randomised trial (*n*=73) comparing ketamine, lidocaine, and dexmedetomidine in a bariatric ERAS program found lidocaine to be superior for pain control and LOS reduction, while ketamine performed the worst, requiring more rescue analgesia and causing more nausea.^15^ Our study also showed an association between ketamine administration and increased rates of PONV, highlighting the potential influence of ketamine’s pro-emetic properties.^3^^,^^4^ In summary, early growing evidence suggests ketamine infusions offer little benefit and may cause harm within low-opioid ERAS protocols, making their inclusion unlikely to improve outcomes such as LOS, opioid consumption, or resource utilisation.

Several variables in our study demonstrated independent associations with both LOS and ICU admission. These findings warrant discussion, as they likely reflect differences in postoperative recovery trajectories between laparoscopic and open surgical approaches. Patient characteristics such as age and BMI may influence the selection of surgical technique. The favourable odds for LOS and ICU admission observed with truncal blocks in our study are consistent with expectations, as this subgroup primarily underwent laparoscopic procedures. In contrast, the more complex open surgeries are typically managed with epidural analgesia.

This study has several limitations. First, it was a single-centre trial focusing on colorectal, ventral hernia, and surgical oncology patients, which may limit generalisability. While a pragmatic design reflects real-world clinical practice, results may not be directly applicable to other institutions with varying ERAS protocols. Future multicentre trials are necessary, though logistical challenges and differences in ERAS implementation may complicate broader coordination. Second, ketamine’s effects in non-abdominal procedures, such as major orthopaedic and spine surgeries, where postoperative pain and opioid use are more pronounced,^16^ warrant further investigation. Third, this study did not include patient-reported outcomes, such as pain scores, emotional well-being, or functional recovery measures. While our pragmatic approach relied on electronic health record-based outcomes, the lack of subjective patient feedback limits our ability to assess the full impact of ketamine on recovery from the patient’s perspective.^17^^,^^18^ Future studies incorporating validated patient-reported outcome measures, such as Patient-Reported Outcomes Measurement Information System (PROMIS)^19^ scores, are needed to address this gap. Fourth, we did not evaluate how ketamine interacted with other multimodal analgesic components, such as paracetamol or ketorolac, which play a crucial role in ERAS pain management.^18^ Given the variability in adjunctive analgesic strategies, future research should explore potential synergistic or antagonistic effects when ketamine is combined with other non-opioid analgesics. Fifth, although multiple statistical tests were performed, we did not adjust for multiple comparisons, increasing the risk of type I error. While our findings align with previous research suggesting limited benefit of ketamine within ERAS protocols, they should be interpreted with caution.

In conclusion, ketamine did not provide additional benefit when included within a multimodal, low-opioid utilisation ERAS protocol for patients undergoing colorectal, ventral hernia, or surgical oncology procedures. While ERAS protocols are effective, the individual contributions of each agent to the overall efficacy of a program remains unknown. As demonstrated here, the benefits of an analgesic adjunct might be outweighed by negative side-effects. Future trials should focus on refining the roles of other key analgesic components in ERAS programmes.

## Authors’ contributions

Study design and planning: BLR, BFSA, MDM, MLD, TWR, MDK

Clinical implementation of study protocol; ERAS program establishment and protocol monitoring: BLR, BFSA, MDM, CGP, SRR

Coordination of nursing and clinician education efforts for the trial, and stewardship of the consent forms: BLR, CGP, SRR

Drafting of manuscript: BLR, YG, HM, TWR, MDK

Review and editing of manuscript: all authors

Designed the clinical informatics design, allowing the trial to operate pragmatically within the electronic medical record: REF, JPW

Trial pharmacist: KHS

Study statistician: YG, LC

Study coordination and management of team processes and regulatory oversight: MLD, HM

Management of the LHS and oversight of funding and operations of the trial through collaboration of this enterprise: TWR

## Funding

The Vanderbilt Institute for Clinical and Translational Research (VICTR) Learning Healthcare System Platform under Clinical Translational Science Award (UL1 TR002243) from the US National Center for Advancing Translational Sciences. Its contents are solely the responsibility of the authors and do not necessarily represent official views of the National Center for Advancing Translational Sciences or the National Institutes of Health (Bethesda, MD, USA).

## Declarations of interest

MDM serves on the scientific advisory board for Takeda Pharmaceuticals, and the role is unrelated to this work. REF owns stock in 3M, and he serves as a consultant for Oak Hill Clinical Informatics and Phillips. He declares no conflicts of interest with this work. TWR has relations with Cumberland Pharmaceuticals, Inc. (Director of Medical Affairs), Cytovale, Inc. (Consultant), and Sanofi, Inc. (Member of the Data Safety and Monitoring Board). No conflicts or relationships are related to this work. All other authors declare that they have no conflicts of interest.

## Figures and Tables

**1:** 

Name	Affiliation
Gordon, Bernard, MD	Vanderbilt Institute for Clinical and Translational Research; Division of Allergy, Pulmonary, and Critical Care Medicine
Robert Dittus, MD, MPH	Vanderbilt Institute for Medicine and Public Health; Division of General Internal Medicine and Public Health; VA Tennessee Valley Healthcare System Geriatric Research, Education and Clinical Center
Peter Embi, MD, MS, FACP, FACMI, FAMIA, FIAHSI	Department of Biomedical Informatics
Robert Freundlich, MD	Department of Anesthesiology, Division of Anesthesiology Critical Care Medicine
Cheryl Gatto, PhD, PMP	Vanderbilt Institute for Clinical and Translational Research; Department of Emergency Medicine
Frank Harrell Jr, PhD	Department of Biostatistics
Paul Harris, PhD, FACMI, FIAHSI	Department of Biomedical Informatics
Tina Hartert, MD, MPH	Division of Allergy, Pulmonary, and Critical Care Medicine; Vanderbilt Institute for Medicine and Public Health
Catherine Ivory, PhD, RN-BC, NEA-BC, FAAN	Nursing Research Administration
Ruth Kleinpell, PhD, RN, FAAN	School of Nursing
Sunil Kripalani, MD, MSc	Division of General Internal Medicine and Public Health
Lee Ann Liska, MBA, RN	Clinical Enterprise Administration
Patrick Luther, MHS	Community Member
Jay Morrison, MSN, RN, CPPS	Nursing Administration (Vanderbilt Hospital and Clinics)
Jill Pulley, MBA	Vanderbilt Institute for Clinical and Translational Research; Division of Allergy, Pulmonary, and Critical Care Medicine
Edward Qian, MD	Department of Biomedical Informatics
Kris Rehm, MD	Pediatric Hospital Medicine
Todd Rice, MD, MSc	Division of Allergy, Pulmonary, and Critical Care Medicine; Vanderbilt Institute for Clinical and Translational Research
Russell Rothman, MD, MPP	Division of General Internal Medicine and Public Health
Patti Runyan, DNP, MBA, RN, NEA-BC	Children’s Hospital Operations
Wesley Self, MD, MPH	Department of Emergency Medicine; Vanderbilt Institute for Clinical and Translational Research
Matthew Semler, MD, MSc	Division of Allergy, Pulmonary, and Critical Care Medicine; Vanderbilt Institute for Clinical and Translational Research
Jennifer Slayton, DNP	Quality, Safety & Risk Prevention
Robin Steaban, MSN, RN, NEA-BC	Nursing Administration (Vanderbilt Hospital and Clinics)
Cosby Stone Jr, MD, MPH	Division of Allergy, Pulmonary, and Critical Care Medicine
Philip Walker, MLIS, MSHI	Eskind Biomedical Library
Consuelo Wilkins, MD, MSCI	Vanderbilt Institute for Clinical and Translational Research, Division of Geriatric Medicine
Adam Wright, PhD	Department of Biomedical Informatics
Autumn Zuckerman, PharmD	Specialty Pharmacy Clinical Services
Chad Fitzgerald, JD	Quality, Safety & Risk Prevention
