# Supplementary material for: IMpact of PerioperAtive KeTamine on Enhanced Recovery After abdominal Surgery (IMPAKT ERAS): a pragmatic randomised single-cluster trial
Source: Br J Anaesth. 2025 Aug 6;135(6):1770–8. doi: 10.1016/j.bja.2025.08.001 (PMC12799394; doi:10.1016/j.bja.2025.08.001)

##### The IMpact of PerioperAtive Ketamine on Enhanced Recovery after AbdominalSurgery (IMPAKT ERAS): A Randomized Controlled Trial

**Online-only Supplemental Material**

**Appendix S1:** Vanderbilt University Medical Center's Enhanced Recovery After Surgery Protocol for colorectal surgery (page 2)

**Appendix S2:** Vanderbilt University Medical Center's Enhanced Recovery After Surgery Protocol for surgical oncology (page 24)

**Appendix S3:** Vanderbilt University Medical Center's Enhanced Recovery After Surgery Protocol for ventral hernia repair (page 32)

**Table S1:** PRECIS-2 scores for trial domains (page 50)

**Table S2:** Conversion factors for various opioids to morphine (page 51)

**Table S3:** Odds of consuming more opioids by randomization arm and covariates (page 53)

**Table S4:** Odds of experiencing an adverse event by randomization arm and covariates (page 54)

**Table S5:** Odds of meeting early discharge milestones by randomization arm and covariates (page 55)

**Table S6:** Interactions between ketamine administration and patient or surgery- related covariates on length of stay (page 56)

**Table S7:** Primary and secondary outcomes for the per-protocol analysis (page 57)

**Table S8:** Post-hoc analysis of various measurements of hospitalization length and readmission rates (page 58)

**Figure S1:** PRECIS-2 wheel (page 59)

**Figure S2:** Schematic illustration of the randomization for the IMPAKT ERAS trial (page 60)

###### Appendix S1: Vanderbilt University Medical Center's Enhanced Recovery After Surgery Protocol for colorectal surgery

**Anesthesia Guidelines for Colorectal Surgery 12.1.2018 1**

**Vanderbilt University Department of Anesthesiology Practice Standardization Protocol for Colorectal Surgery**


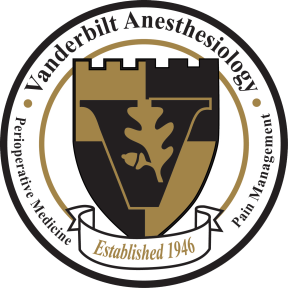


**Management of Patients Undergoing Colorectal Surgery Version 12.1.2018**

**Updates to previous version (12.2017) highlighted**

Category: Perioperative Management

Individuals involved: All Anesthesia care team members who participate in the care of

patients undergoing colorectal surgery

Guidelines applicable to: All patients undergoing general anesthesia for the laparoscopic or open

surgical treatment of colorectal disease.

Reviewed by: This document was developed jointly and reviewed by members of the Colorectal PCC and the MSA Division, including Drs. McEvoy, , Wanderer; Jennifer Jayaram, APRN; and by the colorectal surgeons, including Drs. Geiger, Hopkins, Hawkins, Ford, Muldoon.

Contents:

- Updates to previous version
- Purpose, Targeted Patients, Targeted Length of Stay
- Summary Flowchart p.3
- Perioperative Management
  - Preoperative Interventions p. 4-5

##### Intraoperative Interventions p. 6-8

- - **Postoperative Interventions** p. 9-14
    - Special Circumstances- **Opioid-Tolerant Patients** p.14-16
    - **Ketamine Infusion** Protocol Addendum p.18-20

**2 Anesthesia Guidelines for Colorectal Surgery 12.1.2018**

**Updates to Previous Version (12.2017) Highlighted Below**

Reduced targeted length of stay No routine use of scopolamine patch

Focus on TIVA

Need for 2^nd^ IV insertion for ketamine infusion Methadone intraop dosing changes

Low-carb beverage consumption preoperatively Perioperative Assessments of pain history

I-FEED paradigm for post operative diet advancement Post- operative multimodal analgesics described in more detail Formalized Ketamine infusion inclusion in post operative period

Post-discharge guidance provided Maximizing Multimodal guidance

Timing of sign-off/ when to reconsult Periop Team Guidance for the Opioid Tolerant Patient

**Colorectal Anesthesia Management Guideline**

- **Purpose:**
  - To create a Colorectal Enhanced Recovery After Surgery Program that provides standardization and (where possible) evidence-based guidelines for the anesthetic care of patients undergoing colorectal surgery at Vanderbilt University.
  - To provide perioperative care that maximizes perioperative pain control and reduces morbidity and cost related to cardiac, pulmonary, renal, endocrine, infectious disease, or post -operative nausea and vomiting complications.

##### Target Patients: Colorectal surgery patients

- - **Inclusion Criteria –** all elective colorectal surgical patients, except as noted below.

##### Exclusion Criteria/Contraindications to any specific ERAS component –

- - - Allergies or adverse reactions to any medications listed
- **Targeted length of stay** depending on surgical procedure (H= local hotel as an option after discharge for patients living at a distance)
- Laparoscopic abdominoperineal resection: 3 days
- Open abdominoperineal resection: 3 days
- Laparoscopic low anterior resection: 3 days
- Open low anterior resection: 3+H days
- Laparoscopic sigmoid colon resection: 3 days
- Open sigmoid colon resection: 3+H days
- Laparoscopic total abdominal colectomy: 3+H days
- Open total abdominal colectomy: 3+H days
- Laparoscopic total abdominal proctocolectomy with IPAA: 3+H days
- Open total abdominal proctocolectomy with IPAA: 3+H days
- Laparoscopic total abdominal procotcolectomy: 3+H days
- Open total abdominal proctocolectomy: 3+H days
- Laparoscopic ileocolic resection/right colon resection: 2 days
- Open ileocolic resection/ right colon resection: 3 days
- Ileostomy/ colostomy takedown: 2+H days

**Anesthesia Guidelines for Colorectal Surgery 12.1.2018 3**

Pre-meds Regional

Gabapentin 100-300 mg PO Acetaminophen 1 g PO

Bilateral TAP + Rectus Sheath

vs.

Epidural (for laparotomy) Based on Case

ERAS Protocol: Colorectal

Preop

Colorectal Surgery

Induction Airway Access / Monitors Opioids

ETT

At least 2 IVs Standard ASA monitors

Invasive monitors if needed

BIS if TIVA

Avoid

Give Esmolol for HR control

If on chronic opioids: Methadone 5-10 mg IV

Ad Lib, except:

- Avoid induction opioids
- Give Ketamine 0.5 mg/kg
- Lidocaine 1.5mg/kg
- Esmolol for HR control

Intraoperative

Multimodal Analgesics IV Infusions PONV Fluids

•

Ketorolac 30 mg IV at fascia closure

Ketamine 5 mcg/kg/min until fascia closed THEN 2.5 mcg/kg/min until emergence

- Lidocaine 2 mg/min stop at case end – restart in PACU


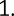

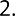

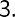

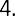


Based on Risk Factors: Ondansetron 4 mg

Dexamethasone 4 mg

Haloperidol 0.5-1 mg Propofol gtt or TIVA

Goal 2-3 mL/kg/hr

LR, Normosol, Plama-Lyte Median 1600 mL/case

Multimodal Analgesics Opioids PONV (PRN)

- Gabapentin
- Acetaminophen
- NSAID
- Ketamine 2.5 mcg/kg/min x 48 hrsif no epidural
- Lidocaine gtt x 24 hrs if no epidural


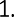

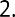


Tramadol 50mg PO Oxycodone 5 mg PO Avoid IV, unless NPO


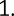

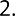

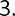

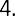


Ondansetron 4 mg IV Haloperidol 0.5-1 mg Scopolamine Patch Promethazine IV

Postop

Adjust all medications based on Age, CrCl, medication sensitivity, chronic opioid use

**4 Anesthesia Guidelines for Colorectal Surgery 12.1.2018**

# Preoperative Management

**Preoperative Oral Intake**

- - Complex carbohydrate drink for all patients – series of 3 drinks: Preop day at 1200, 2000 and DOS 0600
  - Protein supplementation as indicated by nutrition screen
  - Encourage clear liquid PO intake until 2 hours prior to OR time^1,2^
    - Extend to 4 hours prior to OR if history of advanced diabetes/gastroparesis (consult anesthesiologist for questions)

**Conduct or review pain history that includes:**

- - Current opioid and non-opioid pain medications
    - Current opioid provider, and verification via CSMD
  - Sites of pain and baseline pain scores
  - Previous interventions and efficacy
  - Co-morbid psychiatric conditions and medications

**Orders/Interventions written by Perioperative Consult Service**

- **Pre-emptive Analgesia:**
  - **Gabapentin:** 100- 300 mg PO 1 hour before OR time^3^
    - Reduce to 100 mg PO in patients >65y
    - Consider not giving or reducing to 100 mg PO in patients >75y
    - Consider dose reduction in patients with OSA
    - Consider a higher dose in those who are opioid dependent, if not at increased risk for sedation as above
    - For those on home gabapentin, ensure that 100-150% of home dose was taken on AM of surgery (either at home or in preop holding area).
  - **Acetaminophen:** 1000 mg PO 1 hour before OR time
    - Reduce to 650 mg PO if <70kg
    - Don’t use if Child score for Class C liver disease
  - **Postoperative Nausea and Vomiting Prophylaxis**
    - *TIVA preferred for all colorectal surgery patients
    - See CEBA PONV Guidelines on SparkLearn
      - **Principle:** # of PONV prophylaxis agents should equal # of risk factors
    - No routine pre-operative scopolamine for PONV prevention
      - Consider for patients with 4 Apfel Risk Factors (female, non-smoker, h/o PONV/severe motion sickness, ***and*** likely postoperative IV or high dose parenteral opioids) or PONV in PACU

**Anesthesia Guidelines for Colorectal Surgery 12.1.2018 5**

- **Regional Anesthesia (determined by case type)**


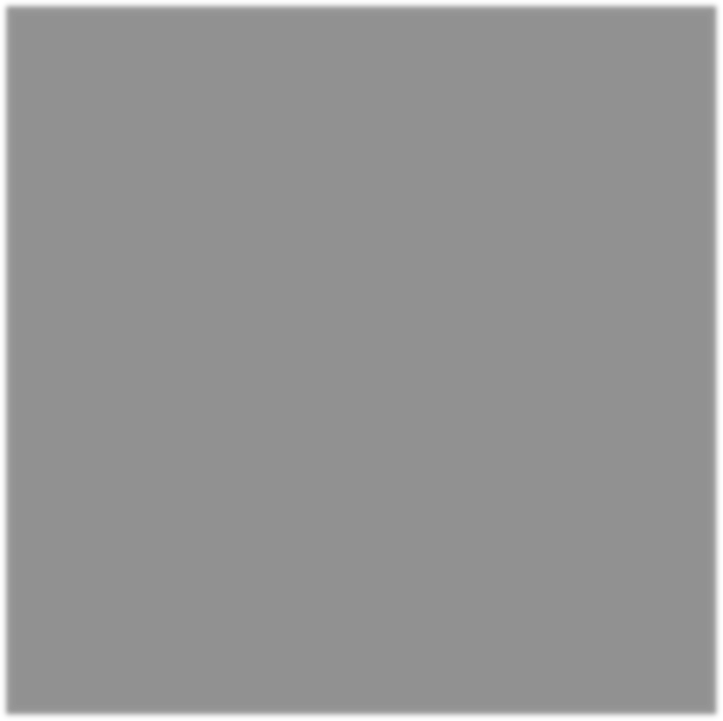

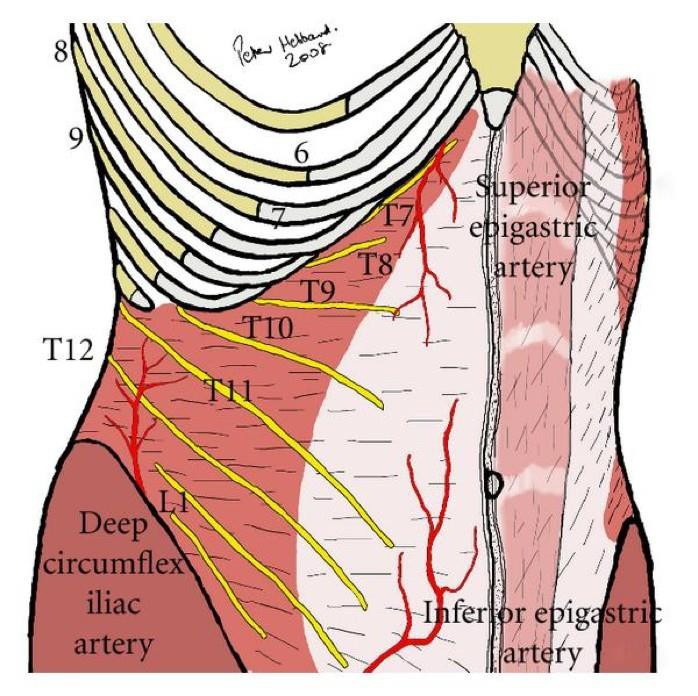

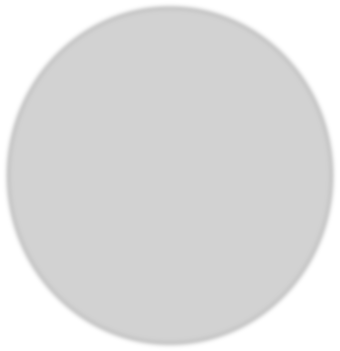

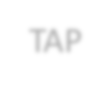

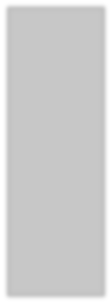

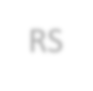

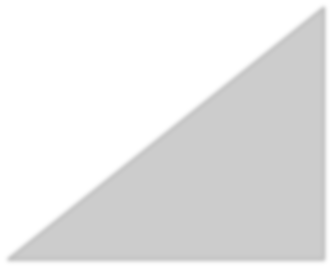

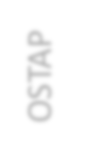


TAP

RS

OSTAP

- **Mini-laparotomy or hand-assisted laparoscopy**
  - Pfannenstiel incision or Periumbilical incision
    - 4 Quadrant blocks (bilateral TAP plus bilat rectus sheath blocks)
      - Ropivacaine 0.25% + dexamethasone 2mg (30-45mL/side total)
- **Ostomy takedown/creation with unilateral incision**
- Perform bilateral TAP Block^4^
  - Ropivacaine 0.25% + dexamethasone 4mg (25-30mL)
  - If Ostomy above umbilicus, consider adding rectus sheath or OS TAP
- **Laparotomy:**
- **Preferred:** Thoracic Epidural^1^ Placement: used if incision is larger than the standard colorectal laparotomy incision (above T8). Discuss with surgeon if TEC is planned. Especially consider if patient opioid dependent.
- If TEC contraindicated: Bilateral TAP plus bilateral rectus sheath blocks (ropivacaine 0.25% + dexamethasone 2mg (30-45 mL/side total)

**6 Anesthesia Guidelines for Colorectal Surgery 12.1.2018**

# Intraoperative Interventions

- - **Total Intravenous Anesthesia with Propofol^5^*** [Please avoid volatile unless propofol allergy]
    - Despite previous use of standard PONV prophylaxis these patients continued to have a high rate of PONV leading to prolonged LOS.
    - Please use a **propofol infusion** as the primary anesthetic agent in these patients.
      - If propofol infusion is >200 mcg/kg/min and the patient is hypertensive or tachycardic, please contact the attending anesthesiologist to discuss management.
      - Consider personally placing IV with or without ultrasound guidance to ensure patency of IV being used for TIVA
    - **Use BIS monitoring initiated pre-induction to reduce risk of intraoperative awareness (ASA recommendation) in any patients who are receiving neuromuscular blockade and not receiving volatile anesthetics^6^**
    - If using volatile agent due to propofol allergy, please contact attending if patient requiring

≥1.3 MAC (end-tidal concentration, adjusted for age) to discuss management approach.

- - **No induction opioids; minimize opioid use during anesthetic^1^**
    - Propofol anesthetic in addition to ketamine and lidocaine
    - Esmolol for heart rate control^7^
    - Anti-hypertensive choice for BP control at discretion of in-room team (e.g. labetalol, hydralazine [currently have a shortage], etc)
  - **If Thoracic Epidural present:**
    - Bolus with 0.125- 0.25% Bupivacaine prior to incision
    - Connect patient to Epidural Infusion Pump upon its arrival to the OR
    - Use during the case as infusion
  - **Ketamine** (for intra-op, use low concentration bag-100mg/100cc)
    - 0.5 mg/kg IV bolus with induction plus 5mcg/kg/min IV after induction until fascia closure.^8^
    - THEN 2.5mcg/kg/min until emergence.^9^
    - Consider reducing (0.25mg/kg) or not using bolus in elderly patients over 65 years of age.
  - **Lidocaine Infusion**
    - 1.5 mg/kg bolus with induction then 2mg/min drip from induction to case end
    - Ordered to continue in PACU in postoperative order set (see postoperative interventions)
    - Contraindications: Unstable heart disease, Recent MI, Heart block, Heart Failure, Electrolyte disturbances, Liver disease, Seizure disorder, Current anti-arrhythmic therapy [e.g. amiodarone, sotalol] ^10^
  - **IV Access:**
    - Lidocaine and ketamine are not able to run on the same IV postoperatively. While under general anesthesia, **please place a second IV** for postoperative access in order to prevent the need for placement awake postoperatively on these patients who may be more difficult access patients.
  - **Ketorolac:** 30 mg IV at fascia closure
    - Reduce to 15 mg IV if >65y, CrCl<30, or patient weight <50kg
    - Consider avoiding for h/o renal dysfunction or GI bleed
  - **Methadone**
    - Please assess patient during emergence for the need for methadone. **Previously, prophylactic methadone was used at induction but the majority of patients do not require opiates during the intraoperative period.**
    - If opioids required, consider methadone on emergence or in PACU (5 mg IV boluses) q5- 10 min prior to using other opioids.
    - **If patient is opioid exposed or tolerant, consider methadone 5-10 mg IV after induction.**

**Anesthesia Guidelines for Colorectal Surgery 12.1.2018 7**

- - **PONV Prophylaxis:** Retching/vomiting in these patients postoperatively may cause disastrous surgical consequences. Consider avoiding sedating anti-emetics in elderly, medically fragile patients, or patients with OSA.
    - Propofol TIVA preferred unless patient has allergy (see intraoperative management)
      - No routine pre-operative scopolamine for PONV prevention, except for high risk patients with 4 Apfel Risk Factors (female, non-smoker, h/o PONV/severe motion sickness, ***and*** likely postoperative IV or high dose parenteral opioids)
      - See Center for Evidenced Based Analgesia (CEBA) PONV Guidelines on SparkLearn
      - **Dexamethasone**: 8mg IV after induction unless given in TAP blocks.
      - **Ondansetron**: 4mg IV given 30 minutes prior to emergence
      - **Haloperidol**: 1mg IV given during skin closure
        - Reduce to 0.5mg IV in elderly patients >65 years of age
        - Current vial concentration is 5mg/mL ****BE CAREFUL WITH DOSING****
        - Do not give in patients with known QTc prolongation
  - Intraoperative Hemodynamic Strategy^1,2,11,12^
    - Do not replace ‘deficit’ empirically
    - Use LR or Plasma-Lyte only: **DO NOT USE NORMAL SALINE**
    - Consider using non-invasive or invasive monitoring for high-risk cardiac patients
      - Maintenance IVF at 2-3mL/kg/hr and then volume boluses as indicated by a dynamic index (e.g. SVV, PPV, PVI, etc). [Median IVF for these cases is 1600 mL)
      - Norepinephrine is preferred vasoconstrictor; can be run through free flowing peripheral IV at low to moderate doses (2-10 mcg/min)^13^
      - Albumin use should follow departmental CEBA Guideline for Intraoperative Albumin Use (**NOTE**: In general, these cases do not qualify for albumin use.)
    - Target UOP ≥0.5mL/kg/hr
      - Note: UOP is dependent on volume status *and* MAP.
  - **Surgical Site Infection Reduction: ‘NO BUGS’ Management Strategy [Outcome: SSI]**
    - **N**ormothermia:^14^
      - Goal core temp >36.0°C
      - Methods: forced air warming blankets, warm all IV Fluids (give no IV in preop), warm room if T<36.0°C
      - Maintenance of normothermia beginning in the holding room with forced air warmer
      - Exceptions – case <30 min in duration or desired hypothermia (CPB, circ arrest, etc)
    - **O**xygenation/Tidal volume:^15^
      - FiO2 ≥ 0.80; TV 6-8mL/kg (IBW) + PEEP ≥6 cm H20 (goal SaO2>93%; consider intermittent recruitment maneuver breaths)^16,17^
        - The most recent meta-analysis showed that there is likely a positive benefit on SSI in colorectal surgical patients when using FiO2 ≥ 0.80^17^
      - Then nasal cannula in PACU to maintain SaO2 94%
      - NC @ 2L/m prior to PACU d/c & for 24 hours postop
    - anti-**B**iotics:
      - Proper drug, proper dose, proper timing (<1 hour of incision, but prior to incision), and proper re-dosing strategy
      - See antibiotic dosing chart in VPIMS. Call OR Pharmacy w/ questions (2-4897)
    - mild **U**nderventilation to normocapnia:^18,19^
      - [These cited studies show no harm to mild hypercapnia, and possible trend toward benefit on SSI. Also, the common practice of hyperventilation should be avoided.]

**8 Anesthesia Guidelines for Colorectal Surgery 12.1.2018**

- - - - **Goal:** ETCO2 >38 mmHg
      - Except for contraindications, such as high ICP, Pulm HTN, etc.
  - **G**lucose control:
    - **Goal**: <150mg/dL (**NOTE:** *DO NOT give dextrose to raise blood glucose unless it is*

*<60mg/dL; no action needed if glucose is <150mg/dL; monitor as below.*) ^20^

- - - **Assessment: Check FSBG on all colorectal patients in Preop**
- if diabetic, check glucose q1hr Intraop
- if non-diabetic and ≥120mg/dL Preop, re-check after 1 hour
  - if ≥120mg/dL, check q1hr during surgery
  - if <120mg/dL, no further re-checks required unless patient took hypoglycemia agent on the day of surgery or initial glucose was < 60 mg/dL
- if non-diabetic and <120mg/dL Preop – no further re-checks
  - - **Management**
- if glucose > 150 mg/dL- Consult attending anesthesiologist to determine need for insulin bolus versus infusion.
- if insulin infusion present at end of case:
  - contact PACU resident and inform of presence of insulin infusion.
  - goal is to transition all patients to Sliding Scale prior to PACU d/c if possible, with goal of <180mg/dL.
  - all diabetic patients to be followed by Diabetic Management Service
  - all non-diabetics leaving PACU on insulin Sliding Scale or infusion to

have Diabetic Management Service consult placed and surgical team to be notified.

- - **S**ite prep:
    - No **s**having and careful use of clippers
    - Keystone CVL Protocol for **s**ite prep on all lines; use CHG prep
    - Use Chloraprep on all CVC and a-line insertion **s**ites
    - Use Chloraprep or Duraprep for surgical **s**ite

- per colorectal surgical protocol, and except for contraindications (e.g. patient allergy/stoma)

- - **Scrub** the hub **-** for accessing all CVLs and PIVs

**Anesthesia Guidelines for Colorectal Surgery 12.1.2018 9**

# Postoperative Interventions

- **Diet and Fluid Management (by primary surgical team)**
  - **Review fluid orders to ensure IVF ≤ 50 mL/hr**
    - **IVF to total 50mL/hr (inclusive of therapeutic medication/infusions like lidocaine/ketamine) on POD 1**
  - **Early Refeeding based on I-FEED Paradigm** (see below)^21^
    - **Advance diet to BRAT diet plus protein* on POD 1 if I-FEED score 0-2 (includes protein shakes)**
    - **Encourage gum chewing and ensure ambulation at least TID starting POD1**


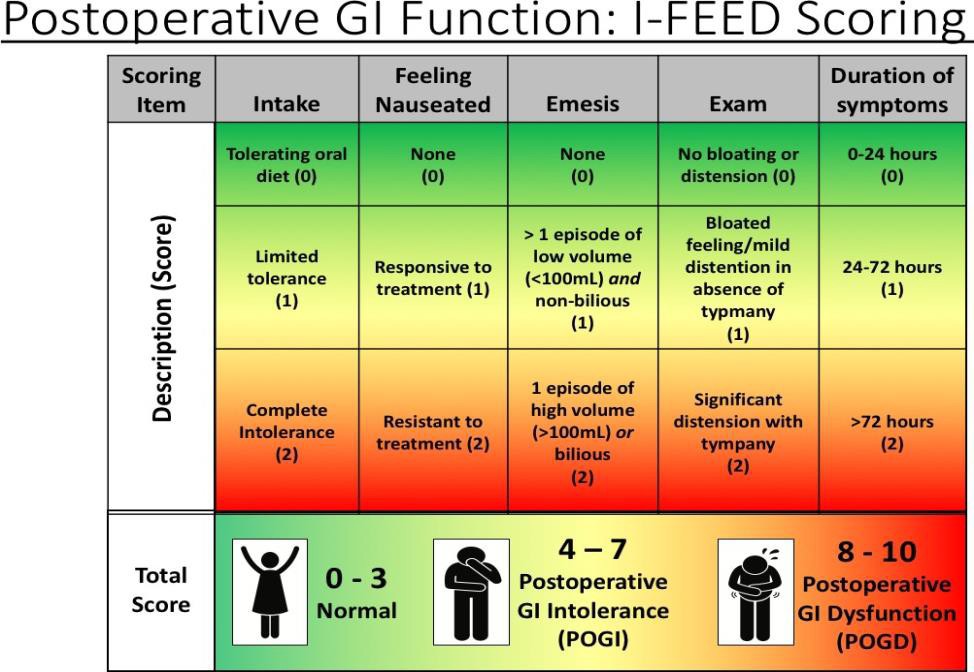

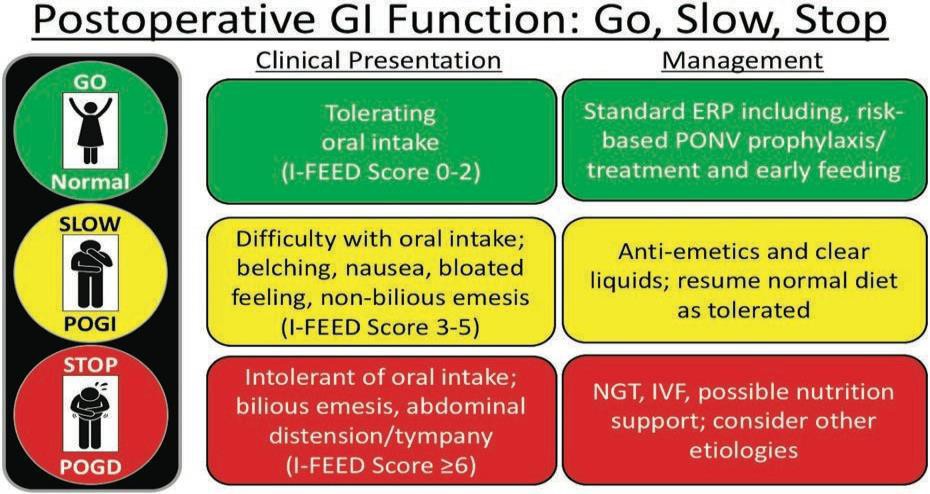


**10 Anesthesia Guidelines for Colorectal Surgery 12.1.2018**

**Postoperative Pain Principles and Algorithm ^22,23^**


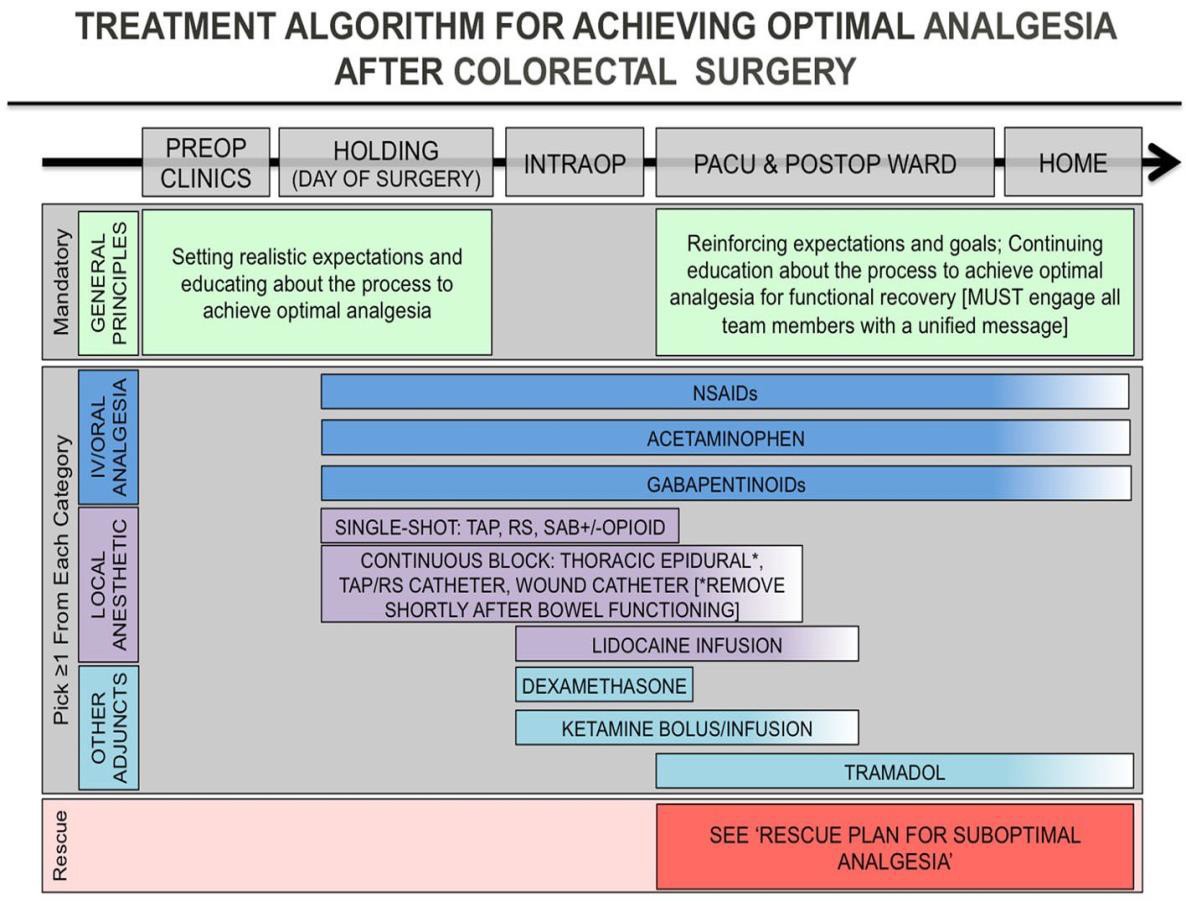


**Orders to be written by Anesthesia Perioperative Consult Service**

- - **Lidocaine Infusion** for 24 hours
    - Continued from PACU **or after TEC removed**
    - Order for PACU to be written by Periop Service for PACU/postop admission phase of care:
      - 1 mg/min IV if <70 kg ~ 15 cc/hr
      - 1.5 mg/min IV if 70-100 kg, ~ 20 cc/hr
      - 2 mg/min IV >100 kg, ~ 30 cc/hr
    - Contraindications: Unstable heart disease, Recent MI, Heart block, Heart Failure, Electrolyte disturbances, Liver disease, Seizure disorder, Current antiarrhythmic therapy [e.g. amiodarone, sotalol] ^10^
    - Can be extended beyond 24 hours if needed based on pain control and PO intake
  - **Ketamine Infusion** for 48 hours^24,25^ (order can be renewed)
    - 2.5mcg/kg/min infusion, using high concentration bag (500mg / 100ml) (not continued from operating room)
    - Requires a 2^nd^ IV line with crystalloid carrier or NS Carrier
- Must order carrier solution when ordering Ketamine (NS at 10cc/hr for carrier)
  - - Use patient’s body weight, up to a max of 100kg. If patient weighs more than 100 kg, use Adjusted Body Weight, up to 100 kg. (Use order-specific weight-based dosing)
    - Contraindications: increased intracranial pressure, history of PTSD or psychotic states, increased intraocular pressure
    - See Ketamine Management Protocol addendum

**Anesthesia Guidelines for Colorectal Surgery 12.1.2018 11**

- - **Gabapentin**
    - 300-600 mg PO q8h starting POD0 until discharge^1^
    - Use lower dose for >65y or if patient having significant sedation/dizziness
    - Gabapentin is cleared renally and dose/frequency should be adjusted based on renal function^26^
- CrCl > 60ml/min: 300-1200mg TID
- CrCl>30-59 ml/min: 200-700mg BID
- CrCl >15-29ml/min 200-700mg once daily
- CrCl < 15- reduce dose in proportion to CrCl ~100-300 mg once daily
- ESRD requiring hemodialysis- dose based on CrCl, plus single supplemental dose of 125-250mg after dialysis
  - - Up titrate dose based on sedation/tolerance if utilizing opioids
    - For Discharge: continue gabapentin x 14 days
- No gabapentin taper unless patient on > 300 mg q8H

o Taper example: 600mg q8h x 7 days, then 300 mg q8H x 7 days

- - **Acetaminophen**
    - 1000 mg PO Q8hr starting POD0 until discharge (max 4000 mg in 24hrs)
- Then 500-1000mg PO Q8h x 3 days and then PRN.
  - - Reduce to 650 mg PO Q6h if <70kg
    - Reduce to 500 mg q 8H for liver disease
- Don’t use if Child Class C liver disease
  - **NSAIDs: ^27-30^**
    - **Ketorolac:** 30 mg IV Q6h x 3 days
- Reduce to 15 mg IV Q6h in patients >65y, CrCl<30, or weight <50kg
  - - Ibuprofen 600 mg q 8H
    - Celebrex 200 mg BID (alternate option)
- In patients >65y, CrCl<30, or weight <50kg - go to 100mg BID
  - **Opioid PRN**
    - Consider lowest possible dose and frequency for pain control
    - Options for opioid naive:
- **First line: Tramadol** 50 mg q 4-6 H PRN (Max 400 mg/24 hrs) , for pain > 4/10.

o Caution with SSRI/SNRI/TCAs due to risk for serotonergic side effects

- **Second line: Oxycodone** 5mg PO Q4 PRN pain >4/10
- Consider Hydromorphone IV PRN bolus for breakthrough pain (only write if pain not controlled with other modalities)
  - - For patients on chronic opioid therapy, ensure meeting 100-125% of home oral dose, with goal of avoiding significant opioid escalation (Perioperative Service should be involved).
- **For complex surgical cases or opioid dependent patients:**
  - **Thoracic Epidural:**
    - Bupivacaine 0.1-0.15% with hydromorphone 10mcg/mL
      - Adjustment to rate and concentration determined by clinical outcomes in pain coverage, hemodynamics - discuss any adjustments with Anesthesiology Attending
      - If Ropivicaine used- start with 0.1% ropivicaine (opioid concentration does not need to change)
    - **Remove TEC if patient has I-FEED score < 3 on evening of POD 2**

**12 Anesthesia Guidelines for Colorectal Surgery 12.1.2018**

o Re-evaluate each day for necessity of TEC, depending on goal length of stay for each

surgical procedure (typically remove TEC day prior to anticipated discharge to ensure adequate pain control on oral regimen

- **Postoperative Nausea and Vomiting (PONV)**
  - **Ondansetron:** 4mg IV/PO Q6hr PRN (write:1^st^ line for nausea and vomiting)
  - **Haloperidol:** 0.5 – 1 mg IV PRN q 4-6 H PRN (write: 2^nd^ line for nausea and vomiting after Zofran)
  - **Add Scopolamine patch (3^rd^ line option only if active PONV despite above)**
    - Consider scopolamine patch preoperatively if 4 or more risk factors for PONV as above.
- Don’t use if >65y and have concern for over-sedation (ex. OSA, dementia) or anti- cholinergic use
- Due to postoperative issues with blurry vision leading to prolonged LOS, scopolamine patch is only to be used in patients with a history of PONV or a history of motion sickness.
- Use only one time dosing, not every 3 days unless persisting PONV
  - **4^th^ line option - Promethazine**: 6.25-12.5 mg IV/PO q 4 H PRN
    - Ensure patent IV prior to promethazine injection as extravasation can cause serious tissue injury
  - **Consider Re-Consulting Perioperative Service by phone or to evaluate if patient’s nausea is intolerable or preventing functional recovery, to consider addition of the following adjuncts:**
  - Alternate options to consider
    - dronabinol (Marinol)- Avoid in patients > 65 yrs
    - dexamethasone (Decadron)– discuss use with surgical team before ordering
    - aprepitant (Emend) - pharmacy must be contacted for special consideration due to cost
- **For patients with pain not controlled with baseline analgesia regimen:**


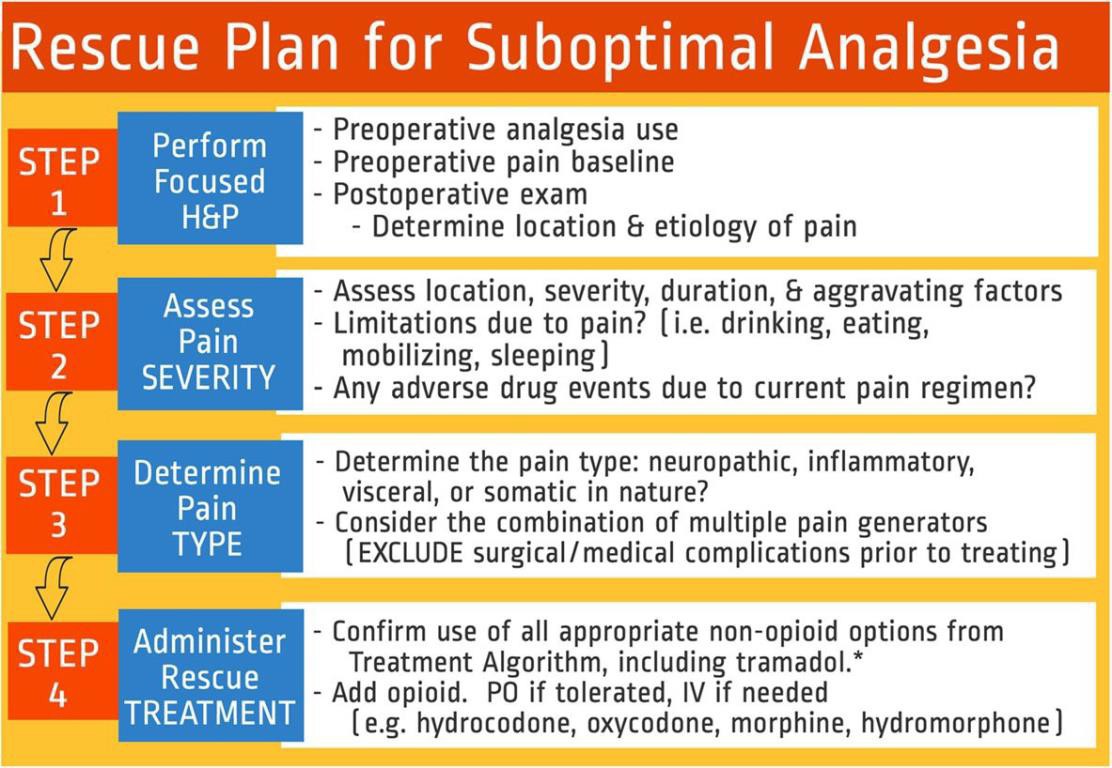


**Anesthesia Guidelines for Colorectal Surgery 12.1.2018 13**

- **Maximodal Analgesia Regimen Options**
  - **Consider Re-Consulting Perioperative Service by phone or to evaluate patient if patient’s pain is intolerable or preventing functional recovery, to consider addition of the following adjuncts**
  - **Other Multimodal Adjuncts** to consider**:**
    - Topical Agents
      - Lidocaine patches
      - Capsaicin
    - Muscle Relaxants
      - Cyclobenzaprine, methocarbamol (available IV, but expensive), baclofen
      - Start with low dose and monitor for sedation, dizziness
      - Stagger dosing with other sedating medications
      - Use with caution in the elderly
      - Avoid if confused, sedated
    - Alpha-2 Adrenergic Agonists
      - Tizanidine, guanfacine
        - Start with low dose and monitor for hypotension, sedation, dizziness
        - Stagger dosing with other sedating medications
        - Helpful for concomitant anxiety, hyperalgesia
        - Use with caution in the elderly
        - Avoid with hemodynamic instability
    - NMDA antagonists
      - Only if not on ketamine infusion
      - Amantadine
      - Dextromethorphan
- Consider **Hydromorphone** PRN bolus if pain control is an issue post-operatively
- 0.25mg IV q 4 H PRN for breakthrough pain if opioid naive, or > 65 yrs old
- 0.5 mg IV q 4 H PRN if opioid tolerant

**Post-discharge Medications Pain Control**

***Guiding principle: Opioids should be tapered first, then multimodal medications***

- **Opioids**
- Adhere to new TN Together Opioid Prescribing Guidelines Opioid Prescription Guidelines
- **Typically would recommend prescribing the opioid medication patient has been utilizing at hospital.**
  - **First line: Tramadol** 50-100 mg q 6 H PRN (Max 400 mg/24 hrs)
    - **Schedule IV and can refill by phone if needed**
    - **Only if no SSRI/SNRI/TCAs**
  - **Second line: Oxycodone** 5mg PO Q6H PRN pain
- Taper to ~ 30-50% dose reduction by 3 days post discharge, then reduced dose q 6 H PRN x 2-3 days, then off
- **Duration of prescription:**
  - Address duration and frequency of opioids in discharge recommendations

*based on patient’s opioid utilization 24 hrs prior to discharge*.

- - **If patient has not used opioids in 24 hrs prior to discharge - consider whether patient needs any opioid, or discharge with only a rescue pack of 3 pills**

**14 Anesthesia Guidelines for Colorectal Surgery 12.1.2018**

- **NSAID:**
  - ibuprofen or celecoxib for 5-7 days
  - Not for inflammatory bowel disease
- **APAP:** max of 3 grams/day for 3 days
- **Gabapentin:**
  - For Discharge: continue gabapentin x 14 days, or throughout opioid taper if prolonged opioid required
    - No gabapentin taper unless patient on > 300 mg q8H
  - Taper example: 600mg q8h x 7 days, then 300 mg q8H x 7 days
- **Other Medications:**
  - **Muscle relaxants**
    - Continue inpatient regimen as PRN x 7 days post op or until off opioids, as long as tolerating without sedation
  - **Alpha-2**
    - Continue inpatient regimen as PRN x 7 days, or until off opioids, as long as tolerating without hypotension, sedation or dizziness
  - **Anti-depressants**
    - Continue any new or dose adjusted medications, but patient will need close follow up with their PCP for monitoring, dose adjustments
- **Nausea Prevention at discharge**
  - Ondansetron 4 mg ODT q 6 H PRN
  - Promethazine PO 12.5 mg q 4 H PRN
  - Scopolamine patch if nausea persisting
  - Dronabinol 5 mg daily if nausea persisting

# Special Circumstances: Opioid-Tolerant Patient

1. Pain History- For patients with chronic pain, ensure/review a thorough pain history can guide their postoperative regimen, and includes:
   1. Current opioid and non-opioid pain medications
   2. Current opioid provider
   3. Verification of current prescriptions via Controlled Substance Monitoring Database (CSMD)
   4. Sites of pain and baseline pain scores
   5. Previous interventions and efficacy
   6. Co-morbid psychiatric conditions and medications)
2. Maximize multimodal therapy before significant increases in opioid dose Consider the addition of additional classes of multi-modal therapy:
   1. Muscle relaxant
   2. Alpha-2 adrenergic agonists
   3. NMDA Receptor antagonists
   4. Antidepressant
   5. Non-pharmacological modalities
3. Psychiatric Comorbidity Management
   1. Ensure adequate management of comorbid psychiatric conditions and ensure utilization of home anxiolytics or antidepressants
   2. Untreated anxiety or depression should be addressed with psychiatry consultation
4. Opioid Management:
   1. Utilize patient’s home opioid medication regimen whenever possible
   2. Ensure baseline daily MME is being met

**Anesthesia Guidelines for Colorectal Surgery 12.1.2018 15**

- 1. Consider increased frequency of PRN opioid before escalating opioid dose or changing opioids
  2. If pain control still an issue, opioid rotation may be warranted
     1. Dose reduction of total MME by 30-50% with opioid rotation to monitor for cross- tolerance

1. At time of discharge: ^31^
   1. Ensure patient has follow up with their opioid prescriber, and ascertain date of follow up.
   2. Adhere to new TN Together Opioid Prescribing Guidelines Opioid Prescription Guidelines
   3. Provide opioid taper guidance back to home opioid regimen
      1. General guidance suggests a dose decrease by ~ 10% of the original dose every 5 to 7 days
   4. May need closer follow up with surgical team.
   5. Chronic pain/opioid tolerant patient will require a prolonged taper off of multimodal therapy as well.
      1. Multimodal regimen should continue throughout opioid taper, and then be tapered back to home regimen, or off
   6. For any patient having difficulty tapering back down to their home regimen, consider referral to Transitional Pain Clinic at OHO

**16 Anesthesia Guidelines for Colorectal Surgery 12.1.2018**

**Vanderbilt Transitional Pain Clinic for Outpatient Pre- and Post-operative**

**Pain Management**

| **Pre-operative Pain Consultation Post-operative Pain Follow-up (with MD) Visit**  Criteria Criteria   - patients on higher dose opioids (≥50 MME) ● only for patients requiring opioids longer   - see chart below than expected by surgeon   - patients on Suboxone   How to Consult How to Consult  ☑ place Ambulatory referral order for **Pain** ☑ surgical service must provide initial discharge  **Medicine** (Ref64) and make a comment for prescription, then if help is needed to further  **pre**-op pain management taper off opioids and control pain, place   - - can be seen same day as Ambulatory referral order for **Pain**   pre-anesthesia clinic (VPEC) **Medicine** (Ref64) and make a comment for  **post**-op pain management  For questions during business | | | | | | | |
| --- | --- | --- | --- | --- | --- | --- | --- |
|  | **Drug** | **Dose** | hours, call: | | | | |
|  | hydrocodone (Vicodin) | ≥50mg per day |  |  |  |  |  |
|  |  |  | (patient is outpatient) **615-** | | **2** | |  |
|  | morphine | ≥50mg per day | (patient is inpatient) **615-** |  | |  |  |
|  | oxycodone | ≥40mg per day | For scheduling assistan | ce contact | |  | |
|  | hydromorphone (Dilaudid) | ≥10mg per day | Location: One Hundred Oaks Clinic | | | | |

**Anesthesia Guidelines for Colorectal Surgery 12.1.2018 17**

**References**

1. Miller TE, Thacker JK, White WD, et al. Reduced length of hospital stay in colorectal surgery after implementation of an enhanced recovery protocol. *Anesthesia and analgesia.* 2014;118(5):1052- 1061.
2. Gustafsson UO, Scott MJ, Schwenk W, et al. Guidelines for perioperative care in elective colonic surgery: Enhanced Recovery After Surgery (ERAS(R)) Society recommendations. *Clinical nutrition.* 2012;31(6):783-800.
3. Schmidt PC, Ruchelli G, Mackey SC, Carroll IR. Perioperative gabapentinoids: choice of agent, dose, timing, and effects on chronic postsurgical pain. *Anesthesiology.* 2013;119(5):1215-1221.
4. De Oliveira GS, Jr., Castro-Alves LJ, Nader A, Kendall MC, McCarthy RJ. Transversus abdominis plane block to ameliorate postoperative pain outcomes after laparoscopic surgery: a meta-analysis of randomized controlled trials. *Anesthesia and analgesia.* 2014;118(2):454-463.
5. Wu ZF, Lee MS, Wong CS, et al. Propofol-based Total Intravenous Anesthesia Is Associated with Better Survival Than Desflurane Anesthesia in Colon Cancer Surgery. *Anesthesiology.* 2018.
6. Zhang C, Xu L, Ma YQ, et al. Bispectral index monitoring prevent awareness during total intravenous anesthesia: a prospective, randomized, double-blinded, multi-center controlled trial. *Chin Med J (Engl).* 2011;124(22):3664-3669.
7. Dhir R, Singh MR, Kaul TK, Tewari A, Oberoi R. Effect of intravenous esmolol on analgesic requirements in laparoscopic cholecystectomy. *Journal of anaesthesiology, clinical pharmacology.* 2015;31(3):375-379.
8. Singh H, Kundra S, Singh RM, Grewal A, Kaul TK, Sood D. Preemptive analgesia with Ketamine for Laparoscopic cholecystectomy. *Journal of anaesthesiology, clinical pharmacology.* 2013;29(4):478- 484.
9. Dallimore D, Anderson BJ, Short TG, Herd DW. Ketamine anesthesia in children--exploring infusion regimens. *Paediatr Anaesth.* 2008;18(8):708-714.
10. McCarthy GC, Megalla SA, Habib AS. Impact of intravenous lidocaine infusion on postoperative analgesia and recovery from surgery: a systematic review of randomized controlled trials. *Drugs.* 2010;70(9):1149-1163.
11. Raghunathan K, Shaw AD, Bagshaw SM. Fluids are drugs: type, dose and toxicity. *Current opinion in critical care.* 2013;19(4):290-298.
12. Kuper M, Gold SJ, Callow C, et al. Intraoperative fluid management guided by oesophageal Doppler monitoring. *BMJ (Clinical research ed).* 2011;342:d3016.
13. Futier E, Lefrant JY, Guinot PG, et al. Effect of Individualized vs Standard Blood Pressure Management Strategies on Postoperative Organ Dysfunction Among High-Risk Patients Undergoing Major Surgery: A Randomized Clinical Trial. *JAMA.* 2017;318(14):1346-1357.
14. Kurz A, Sessler DI, Lenhardt R. Perioperative normothermia to reduce the incidence of surgical-wound infection and shorten hospitalization. Study of Wound Infection and Temperature Group. *The New England journal of medicine.* 1996;334(19):1209-1215.
15. Hovaguimian F, Lysakowski C, Elia N, Tramer MR. Effect of intraoperative high inspired oxygen fraction on surgical site infection, postoperative nausea and vomiting, and pulmonary function: systematic review and meta-analysis of randomized controlled trials. *Anesthesiology.* 2013;119(2):303-316.

**18 Anesthesia Guidelines for Colorectal Surgery 12.1.2018**

1. Yang D, Grant MC, Stone A, Wu CL, Wick EC. A Meta-analysis of Intraoperative Ventilation Strategies to Prevent Pulmonary Complications: Is Low Tidal Volume Alone Sufficient to Protect Healthy Lungs? *Annals of surgery.* 2016;263(5):881-887.
2. Cohen B, Schacham YN, Ruetzler K, et al. Effect of intraoperative hyperoxia on the incidence of surgical site infections: a meta-analysis. *British journal of anaesthesia.* 2018;120(6):1176-1186.
3. Akca O, Kurz A, Fleischmann E, et al. Hypercapnia and surgical site infection: a randomized trial.

*British journal of anaesthesia.* 2013;111(5):759-767.

1. Fleischmann E, Herbst F, Kugener A, et al. Mild hypercapnia increases subcutaneous and colonic oxygen tension in patients given 80% inspired oxygen during abdominal surgery. *Anesthesiology.* 2006;104(5):944-949.
2. McEvoy MD, Wanderer JP, King AB, et al. A perioperative consult service results in reduction in cost and length of stay for colorectal surgical patients: evidence from a healthcare redesign project. *Perioper Med (Lond).* 2016;5:3.
3. Hedrick TL, McEvoy MD, Mythen MMG, et al. American Society for Enhanced Recovery and Perioperative Quality Initiative Joint Consensus Statement on Postoperative Gastrointestinal Dysfunction Within an Enhanced Recovery Pathway for Elective Colorectal Surgery. *Anesthesia and analgesia.* 2018;126(6):1896-1907.
4. McEvoy MD, Scott MJ, Gordon DB, et al. American Society for Enhanced Recovery (ASER) and Perioperative Quality Initiative (POQI) joint consensus statement on optimal analgesia within an enhanced recovery pathway for colorectal surgery: part 1-from the preoperative period to PACU. *Perioper Med (Lond).* 2017;6:8.
5. Scott MJ, McEvoy MD, Gordon DB, et al. American Society for Enhanced Recovery (ASER) and Perioperative Quality Initiative (POQI) Joint Consensus Statement on Optimal Analgesia within an Enhanced Recovery Pathway for Colorectal Surgery: Part 2-From PACU to the Transition Home. *Perioper Med (Lond).* 2017;6:7.
6. Jouguelet-Lacoste J, La Colla L, Schilling D, Chelly JE. The use of intravenous infusion or single dose of low-dose ketamine for postoperative analgesia: a review of the current literature. *Pain medicine (Malden, Mass).* 2015;16(2):383-403.
7. Kator S, Correll DJ, Ou JY, Levinson R, Noronha GN, Adams CD. Assessment of low-dose i.v. ketamine infusions for adjunctive analgesia. *Am J Health Syst Pharm.* 2016;73(5 Suppl 1):S22-29.
8. Hellden A, Odar-Cederlof I, Nilsson G, et al. Renal function estimations and dose recommendations for dabigatran, gabapentin and valaciclovir: a data simulation study focused on the elderly. *BMJ Open.* 2013;3(4).
9. Gaskell H, Derry S, Wiffen PJ, Moore RA. Single dose oral ketoprofen or dexketoprofen for acute postoperative pain in adults. *Cochrane Database Syst Rev.* 2017;5:CD007355.
10. Yu YD, Hwang JH, Seo YE, et al. Effects of Nonsteroidal Anti-Inflammatory Drugs as Patient Controlled Analgesia on Early Bowel Function Recovery after Radical Cystectomy. *Sci Rep.* 2018;8(1):4658.
11. Derry S, Cooper TE, Phillips T. Single fixed-dose oral dexketoprofen plus tramadol for acute postoperative pain in adults. *The Cochrane database of systematic reviews.* 2016;9:CD012232.
12. Derry S, Moore RA. Single dose oral celecoxib for acute postoperative pain in adults. *Cochrane Database Syst Rev.* 2013(10):CD004233.
13. Berna C, Kulich RJ, Rathmell JP. Tapering Long-term Opioid Therapy in Chronic Noncancer Pain: Evidence and Recommendations for Everyday Practice. *Mayo Clin Proc.* 2015;90(6):828-842.

**Anesthesia Guidelines for Colorectal Surgery 12.1.2018 19**

# Ketamine Infusion Protocol- Addendum

1. Background

A low dose Ketamine infusion can be beneficial to improve pain control. This therapy may reduce opioid dose requirements, opioid tolerance, and help reduce post-op nausea and vomiting.

- 1. Ketamine is a dissociative anesthetic agent at high doses, but is an analgesic at low doses–

Ketamine works by blocking -methyl-D-aspartate (NMDA) receptors involved in transmitting pain

- 1. Ketamine acts as an “anti-hyperalgesic” drug and may reduce opioid tolerance
  2. Low-dose continuous infusions are safe and effective for management of postoperative pain, and are associated with less respiratory depression than opioids.
  3. Currently used for a Perioperative Pain management pilot with Enhanced Recovery after Surgery (ERAS) protocols, with possible expansion in future for Acute and Chronic pain patients, pending Pharmacy and Therapeutics Committee Review.

1. Process
   1. Infusion ordered for 48 hours if no thoracic epidural catheter in place by Perioperative Service Attending
   2. Rate of 2.5mcg/kg/min, using high concentration bag (not continued from OR)
   3. Requires a 2^nd^ IV line with crystalloid or Normal Saline carrier
      1. Must order carrier solution when ordering Ketamine (NS at 10cc/hr for carrier)
   4. Use patient’s body weight, up to a max of 100kg. If patient weighs more than 100 kg, use adjusted body weight (calculated within Epic order set) as patient’s weight.
   5. Surgical populations differ on duration of ketamine infusion. See ERAS Protocols.
      1. Bariatric surgery patients: infusion till six AM on POD 1. Adjust duration of infusion in order (i.e. order 16 hours total), and place order comments “Stop ketamine at 6am POD1”
2. Clinical Evidence a.

**20 Anesthesia Guidelines for Colorectal Surgery 12.1.2018**

|  | Adriaenssens et al2 | Subramaniam et al3 | Remerand et al4 | Kim et al5 | Lahtinen et al6 |
| --- | --- | --- | --- | --- | --- |
| Dose Used | - 2.5 mcg/kg/min | - 0.15 mg/kg intraoperative bolus - 2 mcg/kg/min | - 0.5 mg/kg intraoperative bolus - 2 mcg/kg/min | - 0.5 mg/kg bolus + 1 mcg/kg/min infusion - 0.5 mg/kg bolus + 2 mcg/kg/min | - 0.075 mg/kg intraoperative bolus - 1.25 mcg/kg/ min |
| Regimen | Postop only | Intraop + 24 hours postop | Intraop + 24 hours postop | Intraop + 48 hours postop | Intraop + 48 hours postop |
| Ketamine vs Placebo | 15 vs 15 | 15 vs 15 | 75 vs 79 | 18- 1 mcg/kg/min  17- 2 mcg/kg/min  17- placebo | 44 vs 46 |
| Endpoint | Opioid Consumption | Opioid Consumption | Opioid Consumption | Opioid Consumption | Opioid Consumption |
| Results | - 38% opioid reduction at 24 hours and 48% at 48 hours - No change in pain score | - 0% opioid reduction at 24 hours - No change in pain score | - 28% opioid reduction at 24 hours - No change in pain score | - 0% opioid reduction at 48 hours (1mcg/kg/ min) - 42.6% reduction at 48 hours postop (2mcg/kg/min) - No change in pain   score | - 17% opioid reduction at 48 hours postop - No change in pain score |

b.

Clinical Studies- Efficacy1

|  | Clinical Studies- Safety1 | | | | | | |
| --- | --- | --- | --- | --- | --- | --- | --- |
|  |  |  | Adriaenssens et al2 | Subramaniam et al3 | Remerand et al4 | Kim et al5 | Lahtinen et al6 |
|  |  | Dose Used | - 2.5 mcg/kg/min | - 0.15 mg/kg intraoperative bolus - 2 mcg/kg/min | - 0.5 mg/kg intraoperative bolus - 2 mcg/kg/min | - 0.5 mg/kg bolus   + 1 mcg/kg/min infusion   - 0.5 mg/kg bolus   + 2 mcg/kg/min | - 0.075 mg/kg intraoperative bolus - 1.25 mcg/kg/ min |
|  |  | Regimen | Postop only | Intraop + 24 hours postop | Intraop + 24 hours postop | Intraop + 48 hours postop | Intraop + 48 hours postop |
|  |  | Ketamine vs Placebo | 15 vs 15 | 15 vs 15 | 75 vs 79 | 18- 1 mcg/kg/min  17- 2 mcg/kg/min  17- placebo | 44 vs 46 |
|  |  | Safety | Minor events reported:   - Diplopia (2 vs 0) - Dreams (1 vs 1) - Secretion (1 vs 0) - Nausea (1 vs 6) - Vomiting (1 vs 2) - Sedation (0 vs 2) | Minor events reported:   - Headache (0 vs 2) - Dizziness (0 vs 1) - Confusion (1 vs 2) - Hallucinations (1 vs 2) - Excessive Sedation (3 vs 1) - Nausea (3 vs 7) - Motor block (2 vs 4) | Minor events reported:   - Pruritus (23% vs 17%) - Trouble with vision (17% vs 9%) - Nightmares (9% vs 10%) - Pleasant dreams (9% vs 13%) - Hallucinations   (8% vs 11%) | Minor events reported:   - Nausea - Vomiting - Dizziness - Headache - Sedation - No patients experienced bad dreams or hallucinations | Minor events reported:   - Hallucinations (4 vs 0) - Nausea - Vomiting |
|  |  | | | | | | |

1. Assouline et al Meta-analysis
   1. Pain intensity at rest at 24hr was decreased by 32%
   2. Cumulative 24hr morphine consumption was decreased by 28%
   3. Incidence of PONV was decreased by 44%
   4. There was no evidence of a difference in the incidence of respiratory adverse events or hallucination

**Anesthesia Guidelines for Colorectal Surgery 12.1.2018 21**

1. Monitoring:
   1. Nurse monitoring per the PCA/Controlled Substance Infusion Policy
   2. With every new medication, bag, or syringe, and at handover, two qualified, licensed staff members perform an independent double check of the following:
2. Patient name
3. MRN number
4. Medication name and dose
5. Medication concentration
6. Infusion rate
7. Pump settings and guardrails (“Critical Care” vs. “General Care, Pain Management”)
8. Line attachment and clamps open
   1. Nurse monitors vital signs, pain, sedation, and respiration at baseline before infusion, then every 30 min. x2, then every 4 hours after the following, and as needed:
9. Therapy initiation
10. Adverse event or patient deterioration (e.g. change in sedation score)
    1. Respiratory monitoring per policy:
11. Respiratory rate
12. Respiratory quality
13. SPO2
14. Ketamine Failure Management
    1. Ketamine failure defined as severe adverse effects necessitating a discontinuation of ketamine infusion, or intolerable mild adverse effects caused by ketamine that may require dose titration or cessation of infusion.
    2. Any bothersome or persistent adverse effects should be documented, and ordering service (Periop/CPS/APS) should be notified.
    3. Adverse effect management:
       1. Pause ketamine immediately and notify Anesthesia Team and primary surgical team, and call Rapid Response Team if appropriate with:
          1. Acutely altered mental status, confusion, severe unpleasant hallucinations, dysphoria or acute distress, agitation,
          2. Severe tachycardia or hypertension,
       2. Rapid improvement in symptoms expected with cessation of ketamine (20-30 minutes). If symptoms persist, other causes should be determined/evaluated.
          1. For Anesthesia team- evaluate patient, and consider discontinuation of ketamine infusion, and use of low dose benzodiazepine to manage severe adverse effects
          2. Continue frequent reassessment of patient
          3. Consider holding other concurrent deleriogenic medications.
       3. Potential mild adverse effects: mild sedation, blurry vision, dizziness, hypersalivation. Assess for patient tolerance before discontinuing ketamine.
       4. Sedation may be attributable to other causes
          1. Consider holding gabapentin for duration of ketamine infusion
          2. Consider opioid dose adjustment, especially in patients with chronic home opioids
       5. Blurry vision is often attributable to other causes. Consider medication adjustment as follows:
          1. Blurry vision associated with tinnitus, perioral numbness, or distal paresthesias is more likely caused by concurrent lidocaine and lidocaine infusion should be stopped and Anesthesia team notified.

**22 Anesthesia Guidelines for Colorectal Surgery 12.1.2018**

- - - 1. Blurry vision and dizziness, especially with associated dry mouth is most likely concurrent scopolamine patch, and scopolamine patch should be removed if patient is not nauseated. Symptoms from scopolamine patch can take several hours to resolve. Consider holding gabapentin as well with blurry vision associated with dizziness.
      2. Blurry vision with nystagmus is more likely ketamine, and ketamine should be dose titrated down for tolerance.
    1. Hallucinations or nightmares may be severe or mild. Assess patient for tolerance.
       1. Mild hallucinations should not be managed with benzodiazepines, and will resolve with dose titration or discontinuation of ketamine.
       2. Severe hallucinations, causing dysphoria or agitation necessitate cessation of ketamine, and consider use of low dose benzodiazepine.
       3. Consider holding other concurrent deleriogenic medications.
  1. With less bothersome side effects, consider dose decrease, with frequent re-assessment of patient’s symptoms to determine dosing effectiveness and improvement in side effects, as follows:
     1. Decrease infusion to 2.0mcg/kg/min infusion, reassess for adverse effects.
     2. If symptoms persist, decrease infusion to 1.5 mcg/kg/min infusion, reassess for adverse effects.
     3. If symptoms persist, decrease infusion to 1.0 mcg/kg/min infusion, reassess for adverse effects.
     4. If symptoms persist, consider discontinuation of ketamine infusion.
  2. Ketamine order should only be renewed in a patient without intolerable adverse effects, in effort to reduce opioid consumption.

References

1. Jouguelet-Lacoste J, Colla LL, Schilling D, et al. The use of intravenous infusion or single dose oflow-dose ketamine for postoperative analgesia: A review of the current literature. Pain Medicine

2015;16:383-403.

1. Adriaenssens G, Vermeyen KM, Hoffman VL, et al. Postoperative analgesia with IV patient- controlled morphine: Effect of adding ketamine. Br J Anaesth 1999;83:393-6.
2. Subramaniam K, Akhouri V, Glazer PA, et al. Intra- and postoperative very low dose intravenousketamine infusion does not increase pain relief after major spine surgery in patients with preoperative narcotic analgesic intake. Pain Med 2011;12:1276-83.
3. Remerand F, Le Tendre C, Baud A, et al. The early and delayed analgesic effects of ketamine after total hip arthroplasty: A prospective, randomized, controlled, double-blind study. Anesth

Analg 2009;109:1963-71.

1. Kim SH, Kim SI, Ok SY, et al. Opioid sparing effect of low dose ketamine in patients with intravenous patient-controlled analgesia using fentanyl after lumbar spinal fusion surgery.

Korean J Anesthesiol 2013;64(6):524-8.

1. Lahtinen P, Kokki H, Hakala T, et al. S(+)- Ketamine as an analgesic adjunct reduces opioid consumption after cardiac sugery. Anesth Analg 2004;99:1295-301.
2. Kator S, Correll D, Ou JY, et al. Assessment of low-dose i.v. ketamine infusions for adjunctive analgesia. Am J Health-Syst Pharm 2016;73(suppl 1):S22-9.

**Appendix S2: Vanderbilt University Medical Center's Enhanced Recovery After Surgery** 24

**Protocol for surgical oncology**

**Surgical Oncology Anesthesia Guideline 12.2017 1**

**Vanderbilt University Department of Anesthesiology Center for Evidence-Based Anesthesia**

**Evidence-Based Guidance and Practice Protocol**


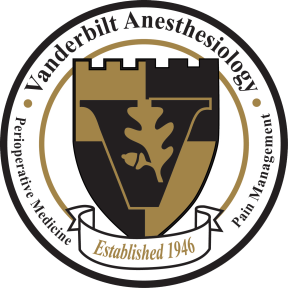


**Management of Surgical Oncology Patients Updates from prior version in red**

Category: Perioperative Management

Individuals involved: All Anesthesia care team members who participate in the

care of patients undergoing CRS and HIPEC

Guidelines applicable to: All patients undergoing general anesthesia for abdominal

cancer resection

Authors: Matt McEvoy, MD

Adam King, MD Kamran Idrees, MD

*Approved Lorri Lee, M.D.*

*Director, Center for Evidence-Based Anesthesia*

**2 Surgical Oncology Anesthesia Guideline**

- - **Purpose:**

#### Surgical Oncology Anesthesia Management Guideline

- - - To provide evidence-based guidelines for the anesthetic care of patients undergoing intra- abdominal surgical oncology procedures at Vanderbilt University.
    - To provide perioperative care that optimizes intravascular fluid management, pain control, surgical site infection reduction, and reduces morbidity and cost related to cardiac, pulmonary, renal, endocrine, infectious disease, or PONV complications.

###### Target Patients: All Surgical Oncology patients undergoing intra-abdominal procedures

- - - **Inclusion Criteria –** all Surgical Oncology patients except as noted below.

###### Exclusion Criteria/Contraindications

- - - - Allergies or adverse reactions to any medications listed;
      - Neuraxial contraindication due to anticoagulation, prior surgery, patient refusal, or anatomy
      - Unique disease or patient condition that may cause specific therapies listed to be contraindicated

**Surgical Oncology Anesthesia Guideline 12.2017 3**

#### Preoperative Interventions:

###### Orders to be written by Perioperative Consult Service

- - - - **Gabapentin:** 100- 300 mg PO 1 hour before OR time[^1^](#_bookmark0)
        - Reduce to 100 mg PO in patients >65y
        - Consider not giving or reducing to 100 mg PO in patients >75y
      - **Tylenol:** 1000 mg PO 1 hour before OR time
        - Reduce to 650 mg PO if <70kg
        - Don’t use if h/o liver disease
      - **Thoracic Epidural** Placement by Perioperative Service

###### Postoperative Nausea and Vomiting Prophylaxis

- - - - - Consider **scopolamine** patch if >2 risk factors for PONV

Don’t use if >65y and have concern for over-sedation (ex. OSA, dementia) or anti-cholinergic use

- - - - - Consider TIVA for patients at high risk for PONV
        - See PONV Guidelines on CEBA Website

**4 Surgical Oncology Anesthesia Guideline**

#### Intraoperative Interventions

###### Please see the end of the document for information about fluid, temperature and hemodynamic management

- - - **No induction opioids; minimize opioid use during anesthetic**[**^2^**](#_bookmark1)
      - Volatile or propofol anesthetic in addition to ketamine
      - Esmolol for heart rate control

###### Thoracic Epidural

- - - - Bolus with 1/8^th^ - 1/16^th^ % Bupivacaine prior to incision
      - Connect patient to Epidural Infusion Pump upon its arrival to the OR

###### Ketamine

- - - - 0.5 mg/kg bolus plus 5mcg/kg/min after induction until fascia closure[.^3^](#_bookmark2)
      - Consider reducing (0.25mg/kg) or not using bolus in elderly patients over 65 years of age.

###### Lidocaine

- - - - 1.5 mg/kg bolus with induction then 2mg/min drip from induction to case end
      - Ordered to continue in PACU in postoperative order set (see postoperative interventions)
      - Contraindications: Unstable heart disease, Recent MI, Heart block, Heart Failure, Electrolyte disturbances, Liver disease, Seizure disorder, Current anti-arrhythmic therapy [e.g. amiodarone, sotalol] [^4^](#_bookmark3)
    - **Ketorolac:** 30 mg IV at fascia closure
      - Reduce to 15 mg IV if >65y, CrCl<30, or patient weight <50kg.
      - Consider avoiding for h/o renal dysfunction or GI bleed

###### Methadone

- - - - Consider methadone 10-20mg IV with induction for patients with chronic opiate use preoperatively; may consider higher doses based on home opioid regimen.
      - If opioids required, consider methadone on emergence or in PACU (5 mg IV boluses) q5-10 min prior to using other opioids.
    - **PONV Prophylaxis:** Wretching/vomiting in these patients postoperatively may cause disastrous surgical consequences. Consider avoiding sedating anti-emetics in elderly, medically fragile patients or OSA patients.

###### Prophylactically use at least two agents from different classes to reduce PONV and consider additional if risk factors > 2

- - - - Consider TIVA for patients at high risk for PONV
        - See Department PONV Guidelines on CEBA Website
      - **Dexamethasone**: 8mg IV after induction unless given in TAP blocks.
      - **Ondansetron**: 4mg IV given 30 minutes prior to emergence

**Surgical Oncology Anesthesia Guideline 12.2017 5**

#### Postoperative Interventions

**Orders to be written by Perioperative Consult Service**

- **Postoperative Pain:**
  - **Thoracic Epidural:**
    - Bupivacaine 0.1% with hydromorphone 10mcg/mL
    - Re-evaluate each day for necessity of TEC, goal for removal 1 day prior to anticipated discharge, to assess PO pain regimen efficacy
    - Remove once patient having active flatus or return of bowel function
  - **Lidocaine Infusion** for 24 hours
    - Continued from PACU or after TEC removed
    - Order for PACU to be written by Periop Service for PACU/Postop admission phase of care:
      - 1 mg/min IV if <70 kg
      - 1.5 mg/min IV if 70-100 kg
      - 2 mg/min IV >100 kg.
    - Contraindications: Unstable heart disease, Recent MI, Heart block, Heart Failure, Electrolyte disturbances, Liver disease, Seizure disorder, Current antiarrhythmic therapy [e.g. amiodarone, sotalol] [^4^](#_bookmark3)

###### Gabapentin

- - - 300-600 mg PO q8 H starting POD0 until discharge
- Then 300 mg PO q8 h x 14 days [2 week post-op course total][^1^](#_bookmark0)
- No gabapentin taper unless patient on > 300 mg q8H

o Taper example: 600mg q8h x 7 days, then 300 mg q8H x 7 days

- - - Use lower dose for >65y or if patient having significant sedation/dizziness
    - Consider change to pregabalin (Lyrica) if patient does not tolerate gabapentin
  - **Tylenol:** 1000 mg PO Q8hr starting POD0 until discharge (max 3000 mg in 24hrs)
- Then 1000mg PO Q8h x 3 days and then PRN.
  - - Reduce to 650 mg PO Q6h if <70kg
  - **Ketorolac:** 30 mg IV Q6h x 3 days
    - Reduce to 15 mg IV Q6h in patients >65y, CrCl<30, or weight <50kg
    - Avoid if significant liver resection
    - Monitor Urine output throughout therapy
  - **Opioid PRN**
    - Consider lowest possible dose and frequency for pain control
    - Options:
- **Tramadol :** 50 mg q 4 H PRN (Max 400 mg/24 hrs) , for pain > 4/10
  - Avoid if patient uses SSRI/SNRI
- **Oxycodone**: 5mg PO Q4 PRN pain >4/10.
- Consider **Hydromorphone** PRN bolus for breakthrough pain
  - 0.5 mg IV q 4 H PRN, for breakthrough pain only
  - 0.25 mg IV q4 H PRN if > 65 y and opioid naïve

**6 Surgical Oncology Anesthesia Guideline**

- - - For discharge: Opioid PRN q 6 H PRN x 3-5 days
    - • First option: Tramadol (Schedule IV and can refill by phone if needed)
    - • Second option: Oxycodone
  - *For patients with chronic pain, utilize patient’s home opioid medication regimen whenever possible. Increase frequency of PRN opioid before escalating opioid dose or changing opioids.
  - Consider **Hydromorphone** PCA if above ineffective, or unable to place TEC

###### Postoperative Nausea and Vomiting

- - - **Ondansetron:** 4mg IV/PO Q6hr PRN

###### First Line Agent

- - - **Haloperidol:** 0.5 – 1 mg IV PRN
    - **Promethazine**: 6.25-12.5 mg IV/PO PRN
      - Ensure patent IV prior to promethazine injection as extravasation can cause serious tissue injury

###### Intraoperative Cardiovascular, Fluid, and Metabolic Management

- - Monitors
    - Place arterial line and central venous line as indicated by patient condition
  - Intraoperative Fluid Strategy
    - Do not replace ‘deficit’ empirically
    - Fluid
      - Crystalloid
        - Use LR or Plasma-Lyte only

###### DO NOT USE ANY NORMAL SALINE

- - - Albumin
- Consider replacing albumin deficit if serum albumin is <2.5G/dL. If used, consider 25% albumin to reduce chloride load. [Give 1G/kg and re-assess; max of 2G/kg/day]
- Discuss any albumin use with attending anesthesiologist prior to use
  - Use PPV/PVI-guided protocol below with to guide fluid and vasopressor management:
    - After induction, give 250mL bolus and track delta SV.
    - If delta SV>+10% with bolus, then repeat.
    - If delta SV<+10% with bolus, then enter maintenance phase.
    - Maintenance IVF: 3mL/kg/hr
    - If MAP<75mmHg and delta SV is <10% after fluid repletion
      - Start phenylephrine infusion up to 0.5mcg/kg/min
      - If hypotension persists after IVF and phenylephrine infusion or if CI<2.5, consult attending anesthesiologist and consider inotrope (norepinephrine, epinephrine, or dobutamine based on consideration of SVRI and CI).
    - If MAP<75mmHg and delta SV >10%, then give 250 mL bolus as above until delta SV

<10% with bolus. [modified from Kuper et al, BMJ 2011]

- - Target UOP ≥0.5mL/kr/hr
    - Intra-abdominal pulse pressure (MAP-IAP) goal >60mm Hg.
    - Use volume or pressors as above to guide during HIPEC portion of case.
    - Note: UOP is dependent on volume status *and* MAP.

**Surgical Oncology Anesthesia Guideline 12.2017 7**

###### Temperature Management

- - Goal core temp >36.5°C and <40°C
    - Patients can *cool* during a long dissection/debulking phase
    - Then, patients can become hyperthermic during HIPEC portion
      - Turn off warmer during HIPEC phase
    - See notes for warming under SSI prevention

###### Optimal Fluid Management [Outcome: AKI, PORF/pulm edema, MACE]

- - Encourage clear liquid PO intake until 2 hours prior to OR time
    - Extend to 4 hours prior to OR time if history of advanced diabetes/gastroparesis (consult anesthesiologist for questions)
  - Intraoperative Fluid Management – see ‘Cardiovascular Management’ above

###### Surgical Site Infection Reduction: ‘NO BUGS’ Management Strategy [Outcome: SSI]

- - **N**ormothermia:
    - Goal core temp >36.5°C
      - Patients can cool during a long dissection/debulking phase
    - Methods: forced air warming blankets , warm all IV Fluids (give no IV in preop), warm room if T<36.5°C
      - Maintenance of normothermia beginning in the holding room with forced air warmer
      - Exceptions – case <30 min in duration or desired hypothermia (CPB, circ arrest, etc)
  - **O**xygenation:
    - FiO2 ≥ 0.3 with PEEP 5cm during case, then nasal cannula in ICU to maintain SaO2

>94%, if extubated in the OR

- - - Ventilate at 6-8mL/kg throughout case using lung protective strategy
  - anti-**B**iotics:
    - Proper drug, proper dose, proper timing (<1 hour of incision, but prior to incision), and proper re-dosing strategy
    - See antibiotic dosing chart in VPIMS. Call OR Pharmacy w/ questions (2-4897)
  - mild **U**nderventilation:
    - **Goal:** ETCO2 38-40 mmHg
    - Except for contraindications, such as high ICP, Pulm HTN, etc.
  - **G**lucose control:
    - **Goal**: 140-180mg/dL (**NOTE:** *DO NOT give dextrose to raise blood glucose unless it is*

*<60mg/dL; no action needed if glucose is 60-140mg/dL; monitor as below.*)

###### Assessment: Check FSBG on all patients in Preop

- if diabetic, check glucose q1hr Intraop
- if non-diabetic and ≥120mg/dL Preop, re-check after 1 hour

- if ≥120mg/dL, check q1hr during surgery

- if <120mg/dL, no further re-checks required unless patient took hypoglycemia agent on the day of surgery or initial glucose was < 60 mg/dL
- if non-diabetic and <120mg/dL Preop – no further re-checks

###### Management

- if glucose >140mg/dL, use Wiz calculator to determine insulin infusion rate.
- if insulin infusion present at end of case:

**8 Surgical Oncology Anesthesia Guideline**

- - contact PACU resident and inform of presence of insulin infusion.
  - goal is to transition all patients to Sliding Scale prior to PACU d/c if possible, with goal of <180mg/dL.
  - all diabetic patients to be followed by Diabetic Mgmt Service
  - all non-diabetics leaving PACU on insulin Sliding Scale or infusion to have endocrine consult placed and surgical team to be notified.
  - **S**ite prep:
    - No **s**having and careful use of clippers
    - Keystone CVL Protocol for **s**ite prep on all lines; use CHG prep
    - Use Chloraprep on all CVC and a-line insertion **s**ites
    - Use Chloraprep or Duraprep for surgical **s**ite
    - **Scrub** the hub **-** for accessing all CVLs and PIVs

1. Schmidt, P. C.; Ruchelli, G.; Mackey, S. C.; Carroll, I. R., Perioperative gabapentinoids: choice of agent, dose, timing, and effects on chronic postsurgical pain. *Anesthesiology* **2013,** *119* (5), 1215-21.
2. Miller, T. E.; Thacker, J. K.; White, W. D.; Mantyh, C.; Migaly, J.; Jin, J.; Roche, A. M.; Eisenstein,

E. L.; Edwards, R.; Anstrom, K. J.; Moon, R. E.; Gan, T. J.; Enhanced Recovery Study, G., Reduced length of hospital stay in colorectal surgery after implementation of an enhanced recovery protocol. *Anesthesia and analgesia* **2014,** *118* (5), 1052-61.

1. Singh, H.; Kundra, S.; Singh, R. M.; Grewal, A.; Kaul, T. K.; Sood, D., Preemptive analgesia with Ketamine for Laparoscopic cholecystectomy. *Journal of anaesthesiology, clinical pharmacology* **2013,** *29* (4), 478-84.
2. McCarthy, G. C.; Megalla, S. A.; Habib, A. S., Impact of intravenous lidocaine infusion on postoperative analgesia and recovery from surgery: a systematic review of randomized controlled trials. *Drugs* **2010,** *70* (9), 1149-63.
3. McEvoy, M. D.; King, A. B.; Wanderer, J.P.; Geiger, T.M.; Tiwari, V.; Terekov, M.; Ehrenfeld, J.M.; Furman, W.R.;Lee, L.A.; Sandber, W.S., A perioperative consult service results in reduction in cost and length of stay for colorectal surgical patients: evidence from a healthcare redesign project. *Perioperative Medicine* **2016,** *5* (3). DOI 10.1186/s13741-016-0028-1
4. Scott, M.J.; McEvoy, M.D.; Gordon, D.B.; Grant, S.A.; Thacker, J.K.M.; Wu, C.L.; Gan, T.J.; Mythen, M.G.; Shaw, A.D.; Miller, T.E., American Society for Enhanced Recovery (ASER) and Perioperative Quality Initiative (POQI) Joint Consensus Statement on Optimal Analgesia within an Enhanced Recovery Pathway for Colorectal Surgery: Part 2—From PACU to the Transition Home. *Perioperative Medicine,* **2017,** 6 (7). DOI 10.1186/s13741-017-0063-6

##### Appendix S3: Vanderbilt University Medical Center's Enhanced Recovery After Surgery 32

**Protocol for ventral hernia repair**

**Ventral Hernia Repair Anesthesia Guideline 7.7.2021 1**

###### Vanderbilt University Department of Anesthesiology Evidence-Based Guidance and Practice Protocol


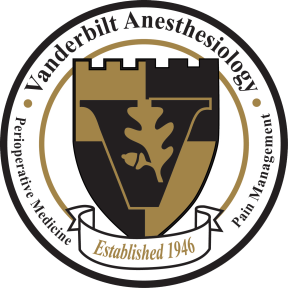


**Management of Patients Undergoing Ventral Hernia Repair 7.7.2021 Updates to previous Version (12.2017) highlighted**

Category: Perioperative Management

Individuals involved: All Anesthesia care team members who participate in the care of

patients undergoing simple, robotic and complex ventral hernia repair

Guidelines applicable to: All patients undergoing general anesthesia for the laparoscopic or

open repair of ventral hernias who will have an inpatient admission

This document was developed jointly and reviewed by Britany Raymond, Emilee Borgmeier, Sydney Ruble, Jennifer Jayaram, Matt Fosnot, and Matt McEvoy; and by surgeons Dr. Broucek and Bradley

###### Updates to Previous Version Highlighted Below

**Preoperative:**

- Low-carb beverage consumption preoperatively
- Surgical incision/approach identification by Anesthesia Consult request, with requested block specified

- **Thoracic epidurals are not routine** for open complex repairs. Consider based upon patient history, such as for opioid-tolerant patients, those with COPD, or concurrent fistula repair

- No routine use of scopolamine patch
- Pre-op medications written by **surgery** team (acetaminophen, gabapentin, celecoxib*)
- Assessment of pain history by Periop Team for those cases requesting involvement post op

###### Intraoperative:

- Methadone intra-op dosing changes
- No ketorolac (preoperative celecoxib)
- Inclusion of ASPIRE intraoperative care standards

###### Postoperative:

- Routine use of ketamine post-op for complex hernia
- Addition of routine use of zanaflex PRN
- Post-operative multimodal analgesics described in more detail
- Post-discharge guidance provided
- Timing of sign-off/ when to consult/re-consult Periop Team

**2 Ventral Hernia Repair Anesthesia Guideline**

###### Ventral Hernia Repair Anesthesia Management Guidelines

- **Purpose:**
  - To provide evidence-based guidelines for the anesthetic care of patients undergoing ventral hernia surgery at Vanderbilt University^1-8^[.](#_bookmark4)
  - To provide perioperative care that maximizes perioperative pain control and reduces morbidity and cost related to cardiac, pulmonary, renal, endocrine, infectious disease, PONV, or surgical site infection complications.
  - Targeted length of stay of 2 nights, discharge by postoperative day 3 for complicated hernias. Others simpler hernia repairs anticipate shorter length of stay depending on complexity of hernia
- **Target Patients: abdominal hernia surgery patients.**
  - **Inclusion Criteria –**
  1. **All cases:**
     - Surgical team will order Periop Team consult with anticipated incision and block requested in clinical comments
     - Preoperative medications to be written by surgery team
     - Ordering of postoperative medications is dependent upon the type & complexity of surgery, as specified below

| **Surgery** | **Expected Stay** | **CPT Codes** | **Pre-Op Meds** | **Post-Op Meds** |
| --- | --- | --- | --- | --- |
| Simple (laparoscopic or  robotic abdominal/ventral | Same day  surgery | 49652, 49653, 49654, 49655,  49656, 49657, 49659 | Surgery | Surgery (consult PCS if  needed due to chronic |
| hernia) |  | + s2900 (Robotic) |  | pain) |
| Open or Robotic incisional  hernia | 1-2 nights | 49540, 49560, 49561, 49565,  49566, 49570, 49572, 49590 | Surgery | Periop Team- Lidocaine,  Oral medications and N/V |
|  |  | + S2900 (Robotic) |  | prophylaxis, +/- Ketamine if |
|  |  |  |  | needed for poor pain |
|  |  |  |  | control |
| Complex Hernia repair  (myocutaneous flap, enterocutaneous fistula  repairs, parastomal | 2-3 nights | 15734, 44346 | Surgery | Periop Team – Lidocaine,  Ketamine, & Oral medications and N/V  prophylaxis |
| hernia) |  |  |  |  |

###### Exclusion Criteria -

**a. Inguinal hernia repairs**

**Ventral Hernia Repair Anesthesia Guideline 7.7.2021 3**

Pre-meds Regional

Bilateral TAPS

+/- OS TAP blocks pending surgical incision

By Surgery Service : Gabapentin 100-300 mg PO Celebrex 100-200 mg PO Acetaminophen 650mg-1 g PO

ERAS Protocol:

Preop

Simple Hernia Repair (Same Day Surgery)

Induction Airway ASPIRE Access / Monitors Opioids

Ad Lib, except:

- Avoid induction opioids
- Give Ketamine 0.5 mg/kg
- Lidocaine 1.5mg/kg
- Esmolol for HR control

ETT

Principles for intra-operative care per MPOG should always be followed

Multimodal Analgesics IV Infusions PONV Fluids

- Standard ASA monitors
- Invasive monitors if needed
- BIS /sedline if TIVA

Avoid

Give Esmolol for HR control

If on chronic opioids: Methadone 5-10 mg IV

Intraoperative

No routine ketorolac unless celecoxib was not given preoperatively

- Ketamine 5 mcg/kg/min until fascia closed THEN

2.5 mcg/kg/min until emergence

- Lidocaine 2 mg/min stop at case end

Based on Risk Factors:

1. Ondansetron 4 mg
2. Dexamethasone 4 mg
3. Haloperidol 0.5-1 mg
4. Propofol gtt or TIVA

Goal 2-3 mL/kg/hr

LR, Normosol, Plama-Lyte Median 1600 mL/case

Multimodal Analgesics Opioids PONV (PRN)

By surgical team:

- Gabapentin
- Acetaminophen
- NSAID + PRN Muscle relaxant

* PERIOP Consult-Lidocaine gtt if chronic pain or poorly controlled post op pain

1. Oxycodone 5 mg PO Avoid IV, unless NPO

1. Ondansetron 4 mg IV
2. Haloperidol 0.5-1 mg
3. Scopolamine Patch
4. Promethazine IV

Postop

Adjust all medications based on Age, CrCl, medication sensitivity, chronic opioid use

ERAS Protocol:

### Complex Ventral Hernia Or Rives Stoppa Repair

Pre-meds Regional

By Surgery Service : Gabapentin 100-300 mg PO Celebrex 100-200 mg PO Acetaminophen 650mg-1 g PO

Type requested by surgical team consult:

4Q truncal blocks Epidural only for extenuating patient factors

Preop

Induction Airway ASPIRE Access / Monitors Opioids

ETT

At least 2 IVs Standard ASA monitors

Invasive monitors if needed

BIS/sedline if TIVA

Give Esmolol for HR control

If on chronic opioids: Methadone 5-10 mg IV

Ad Lib, except:

Avoid induction opioids Give Ketamine 0.5 mg/kg Lidocaine 1.5mg/kg Esmolol for HR control


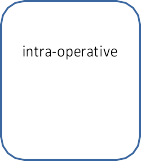


Principles for

care per MPOG should always be followed

Intraoperative

Multimodal Analgesics IV Infusions PONV Fluids

No routine ketorolac unless celecoxib was not given preoperatively

Ketamine 5 mcg/kg/min until fascia closed Lidocaine 2 mg/min stop at case end – restart in PACU

Based on Risk Factors: Ondansetron 4 mg

Dexamethasone 4 mg

Haloperidol 0.5-1 mg Propofol gtt or TIVA

Goal 2-3 mL/kg/hr LR, Normosol, Plama- Lyte preferred

Median 1600 mL/case

By Periop Service: Gabapentin Acetaminophen NSAID

PRN tizanidine

Lidocaine gtt x 24 hrs if no epidural

Ketamine gtt x 48 hrs

Oxycodone 5 mg PO Avoid IV, unless NPO

Ondansetron 4 mg IV Haloperidol 0.5-1 mg Scopolamine Patch Promethazine IV

Multimodal Analgesics Opioids PONV (PRN)

Postop

Adjust all medications based on Age, CrCl, medication sensitivity, chronic opioid use

**4 Ventral Hernia Repair Anesthesia Guideline**

#### Preoperative Interventions:

###### Periop Service to conduct or review pain history that includes:

- - Sites of pain and baseline pain scores
  - Current opioid and non-opioid pain medications
    - Opioid prescriber and verification via CSMD
  - Previous interventions and efficacy
  - Co-morbid psychiatric conditions and medications

###### Orders/Interventions written by Surgery Service: Pre-emptive Analgesia[^6-8^](#_bookmark5):

- - - Acetaminophen[^9^:](#_bookmark7) 1000 mg PO 1 hour before OR time
      - Reduce to 650 mg PO if <70kg
      - Don’t use if Child score for Class C liver disease
    - Gabapentin: 100- 300 mg PO 1 hour before OR time[^10,^](#_bookmark8) [^11^](#_bookmark9)
      - Reduce to 100 mg PO in patients >65y
      - Consider not giving or reducing to 100 mg PO in patients >75y
      - Consider dose reduction in patients with OSA
      - Consider a higher dose in those who are opioid dependent, if not at increased risk for sedation as above
      - For those on home gabapentin, ensure that 100-150% of home dose was taken on AM of surgery (either at home or in preop holding area).
    - Celecoxib 100-200mg PO 1 hour before OR time[^9^](#_bookmark7)^,^ [^12^](#_bookmark10)
      - Consider avoiding if CrCl<30
      - Consider dose reduction if patient weight <50kg
      - Don’t use if recent MI or cardiac surgery
      - Don’t use if Child score for Class C liver disease

###### Postoperative nausea and vomiting prophylaxis

- - - Consider TIVA for PONV management
    - See CEBA PONV Guidelines on SparkLearn
      - Principle: # of PONV prophylaxis agents should equal # of risk factors
    - No routine pre-operative scopolamine for PONV prevention
      - Consider for patients with 4 Apfel Risk Factors (female, non-smoker, h/o PONV/severe motion sickness, *and* likely postoperative IV or high dose parenteral opioids) or PONV in PACU

###### Regional Anesthesia

- - - Surgeon will specify the anticipated surgical incision and request a regional block in the case booking, depending on complexity of case and surgical incision/port sites planned
    - Bilateral TAP plus bilateral rectus sheath blocks
      - Use for robotic ventral or robotic incisional hernia repairs
      - Ropivacaine 0.25% + dexamethasone 2mg (10-15mL/side)
    - Thoracic Epidural (TEC) – not to be placed routinely. Must specifically requested by surgeon consult, or upon discussion with surgical team based upon risk factors below:
      - Complex pain history, opioid tolerance
      - If concurrent fistula repair

**Ventral Hernia Repair Anesthesia Guideline 7.7.2021 5**

- - - - PMH of COPD or other respiratory disease
      - Surgical incision near diaphragm, subcostal hernia, or subxyphoid hernia
      - *Clinical Rationale:* Epidurals are still effective in setting of ERAS/multimodal specifically[^8^.](#_bookmark6) However, they may prolong LOS due to decreased ambulation and urinary retention[^13,^](#_bookmark11) [^14^.](#_bookmark12) Alternatively, TAPs have also been shown to be effective in ventral hernia and could be a reasonable alternative[^13^.](#_bookmark11) Recommendation –TAPs routinely. Consider epidurals for rescue in PACU, patients with chronic pain, COPD, complex repairs with surgeon’s discretion, or anesthesiologist request.

## Intraoperative Interventions

###### Anesthetic Choice:

- - - *May use GETA with Volatile or TIVA with Propofol* for the primary anesthetic

###### Use Processed EEG (Sedline) monitoring initiated pre-induction to reduce risk of intraoperative awareness (ASA recommendation) in any patients who are receiving neuromuscular blockade and not receiving volatile anesthetics[.^15^](#_bookmark13)

- - - If using propofol at >200 mcg/kg/min (or volatile at >1.3 MAC (ET concentration) and patient is hypertensive, please contact attending in order to discuss management approach.
    - ***TIVA preferred** for patients with **high risk** of PONV (Apfel score ≥3)
  - **ERAS Analgesic Management & Opioid Minimization Strategies**
    - **VUMC ERAS Goal: Intraoperative MME *<15***
    - **No induction opioids; minimize opioid use during anesthetic**[**^16^**](#_bookmark14)
      - Esmolol for heart rate contro[l^17^](#_bookmark15) (goal HR<100 in most patients, although degree of CAD and presence of valve disease should be used to determine target)
      - Anti-hypertensive choice for BP control at discretion of in-room team (e.g. hydralazine, etc.)

###### If Thoracic Epidural present:

- - - - Bolus with 0.125% Bupivacaine (or 0.2% Ropivacaine) prior to incision
      - Connect patient to Epidural Infusion Pump upon its arrival to the OR

###### Use during the case as infusion (typically 6-8 mL/hr)

- - - **IV Access:**
      - Lidocaine and ketamine are not able to run on the same IV postoperatively, but this is permitted intraoperatively. While under general anesthesia, **please place a second IV** for postoperative access in order to prevent the need for placement awake postoperatively on these patients who may be more difficult access patients, especially for those patients being admitted.

###### Ketamine[^18,^](#_bookmark16) [^19^](#_bookmark17)

- - - - 0.5 mg/kg IV bolus with induction plus 5mcg/kg/min IV after induction until fascia closure.
      - Consider reducing (0.25mg/kg) or not using bolus in elderly patients over 65 years of age.
        - Use of low-dose midazolam (1-2 mg IV) is not contraindicated, however should not be given routinely to patients
      - Ordered to continue in PACU in postoperative order set (see postoperative interventions)
      - Resume immediately on arrival to PACU in patients who will have postoperative infusion

**6 Ventral Hernia Repair Anesthesia Guideline**

###### Lidocaine Infusion[^20-22^](#_bookmark18)

- - - - 1.5 mg/kg bolus with induction then 2 mg/min drip from induction to case end
      - Contraindications: unstable heart disease, recent MI, heart block, heart failure, electrolyte disturbances, liver disease, seizure disorder, current anti-arrhythmic therapy [e.g. amiodarone, sotalol] [^23^](#_bookmark21)
      - Resume immediately on arrival to PACU in patients who will have postoperative infusion

###### Ketorolac- (only if no pre-op Celecoxib):

- - - - IF needed: consider 30 mg IV at fascia closure
        - Reduce to 15 mg IV if >65y, CrCl<30, or patient weight <50kg
        - Consider avoiding for h/o renal dysfunction, GI bleed; do not give if patient has persistent hypotension

###### Methadone

- - - - Please assess patient during and after emergence for the need for methadone.
      - If opioids are required, consider methadone on emergence or in PACU (5 mg IV boluses) q5-10 min prior to using other opioids.
        - Consider reduction to 2.5mg in elderly patients (Age > 65)

###### If patient is opioid exposed or tolerant, consider methadone *10-20mg* IV after induction. Suggest dose range of 0.15mg/kg IBW (ambulatory surgery) to 0.3 mg/kg IBW for admitted patients[^24^](#_bookmark22)^,^ [^25^](#_bookmark23)

- - **Surgical Site Infection Reduction: ‘NO BUGS’ Management Strategy [Outcome: SSI]**
    - **N**ormothermia[:^26^](#_bookmark24)

o As above with ASPIRE Goals for Temperature metric

- - - **O**xygenation/Tidal volume[:^27^](#_bookmark25)
      - TV 6-8mL/kg (IBW) plus PEEP ≥4 cm H20 throughout case
      - FiO2≥0.3 to keep SaO2≥94% is now considered sufficient in all cases per current literature[^28,^](#_bookmark26) [^29^](#_bookmark27)
      - Then nasal cannula in PACU to maintain SaO2 ≥94%
    - anti-**B**iotics:
      - Proper drug, proper dose, proper timing (≤1hr prior to incision – must be in 3-5 minutes prior to incision), and proper re-dosing strategy
      - See antibiotic dosing chart in i-Care Guidelines. Call OR Pharmacy w/ questions (2-4897)
    - mild **U**nderventilation to normocapnia[:^30,^](#_bookmark28) [^31^](#_bookmark29)
      - **Goal:** ETCO2 >35-40 mmHg, except for contraindication such as high ICP, Pulm HTN, etc.
      - The common practice of hyperventilation should be avoided (e.g. ETCO2 ≤34 mmHg).
      - Studies show no harm to mild hypercapnia, and possible trend toward benefit on SSI.
    - **G**lucose control:^13,14^
- Refer to VUMC Perioperative Glucose Management Protocol for full assessment and management details (P.Henson 2021).
- **Perioperative Glucose Goal**: glucose maintained between 140-180mg/dL (**NOTE:**

*no action needed if glucose is 70-140mg/dL; monitor as below.*)

o Goal is to transition all patients to Sliding Scale prior to PACU d/c if possible, with goal of <180mg/dL.

###### Assessment: Check fasting blood glucose level on all patients in pre-op holding if case duration expected to be longer than 2 hours.

**Ventral Hernia Repair Anesthesia Guideline 7.7.2021 7**

- if diabetic, check glucose q1hr Intraop
- if non-diabetic and ≥120mg/dL Preop, re-check after 1 hour
  - - **S**ite prep:
- No **s**having and careful use of clippers
- Keystone CVC Protocol for **s**ite prep on all lines; use CHG prep
- Use Chloraprep on all CVC and a-line insertion **s**ites
- Use Chloraprep or Duraprep for surgical **s**ite, except for contraindications (e.g patient allergy/stoma)
  - - **Scrub** the hub **-** for accessing all CVCs and PIVs

***As a component of ERAS and care standardization, care in compliance with MPOG ASPIRE Metrics is considered best practice.* Provider adherence to the following metrics will be reported via ASPIRE Task Force. Full text available at ASPIRE METRICS**

- - - **Acute Kidney Injury- https://spec.mpog.org/Spec/Public/18**
      - **Goal:** Hypotension avoidance, BP monitoring (See Blood Pressure components goals below), euvolemia.

###### Blood Pressure Goals & Metrics:

- - - - BP-01- https://spec.mpog.org/Spec/Public/11
        - Goal: Hypotension avoidance, (MAP >55mmHG throughout case).
      - BP-02- https://spec.mpog.org/Spec/Public/12
        - Goal: Regular monitoring, ( ≤10-minute gap in measurement interval).
      - BP-03- https://spec.mpog.org/Spec/Public/34
        - Goal: Hypotension avoidance, (MAP >65mmHG throughout case).
- ***ERAS Goal for MAP > 65mmHG throughout case***

###### Fluids - https://spec.mpog.org/Spec/Public/15 & VUMC Hemodynamic Strategy[^16,^](#_bookmark14) [^32-^](#_bookmark30) [35](#_bookmark30)

- - - **Goal:** avoid the use of colloids and high-chloride solutions in most instances.
    - Strategies:
      - Do not replace ‘deficit’ empirically;
        - Goal: maintain euvolemia intraop (3-4 mL/kg/hr)
      - LR or Normosol/Plasma-Lyte A is preferred
      - Consider using non-invasive or invasive monitoring for high-risk cardiac patients
      - Norepinephrine is preferred vasoconstrictor; can be run through free flowing peripheral IV at low to moderate doses (2-10 mcg/min)[^36-38^](#_bookmark31); if higher doses are needed, consider whether additional IVF is needed or consider placement of CVC
  - **Glucose Management Goals:** *For all patients with and without diagnosis of diabetes, the following metrics represent the minimum threshold of provider compliance expected. Please refer to above glucose management strategies and VUMC Perioperative Glucose Management Protocol:*
    - GLU-01- https://spec.mpog.org/Spec/Public/5
      - Goal: intra-operatively, all Glucoses > 200 receive insulin or recheck within 90 minutes.
    - GLU-02- https://spec.mpog.org/Spec/Public/6
      - Goal: intra-operatively, all glucoses < 60 receive glucose or dextrose or recheck within 90 minutes.
    - GLU-03- https://spec.mpog.org/Spec/Public/38
      - Goal: Perioperatively, all glucose> 200 receive insulin or recheck within 90 minutes.
    - GLU-04:- https://spec.mpog.org/Spec/Public/39

**8 Ventral Hernia Repair Anesthesia Guideline**

o Goal: Perioperatively, all glucoses< 60 receive glucose or dextrose, or recheck within 90 minutes.

- - - GLU-05- https://spec.mpog.org/Spec/Public/43
      - Goal: Perioperatively, all glucoses > 200 receive administration of insulin within 90 minutes

###### Strategies:

- - - - Refer to VUMC Perioperative Glucose Management Protocol.
  - **Medication Overdose** https://spec.mpog.org/Spec/Public/13
    - **Goal:** For all cases where opioids or benzodiazepines administered, administration of naloxone or flumazenil was not required for the case.

###### Myocardial Injury Goals:

- - - CARD-02- https://spec.mpog.org/Spec/Public/27
      - No elevation in troponin within 72 hrs of surgery.
    - CARD-03 - https://spec.mpog.org/Spec/Public/31
      - For high risk surgeries or high risk patients, no elevation in troponin within 72 hrs after surgery.

###### Neuromuscular Blockade Monitoring & Reversal-

- - - **Goals:** From Anesthesia Start to earliest extubation:
      - NMB-01- https://spec.mpog.org/Spec/Public/1: Documentation of a Train of Four count (1, 2, 3, or 4), sustained tetany, or TOF ratio provided by acceleromyography AFTER last dose or stopping of infusion of neuromuscular blocker and before earliest extubation. Note: A Train of Four value of ‘0’ is accepted for cases in which Sugammadex is administered for reversal.
      - NMB-02- https://spec.mpog.org/Spec/Public/2: Administration of Neostigmine or Sugammadex before extubation for cases with non- depolarizing neuromuscular blockade.

###### Prevention of nausea & vomiting

- - - **Goals:**
      - PONV-01- https://spec.mpog.org/Spec/Public/24
        - Patient receives combination therapy consisting of at least two prophylactic pharmacologic anti-emetic agents of different classes preoperatively or intraoperatively.
      - PONV-03- https://spec.mpog.org/Spec/Public/33
        - Patient does not report nausea, have an emesis event or receive an antiemetic during the immediate postoperative period PACU- 6hrs post op.
    - Strategies:
      - Avoid/minimize sedating anti-emetics in elderly, medically fragile patients, or OSA patients.
      - See Center for Evidence Based Anesthesia Post-Operative Nausea and Vomiting Guidelines:
- **Dexamethasone**: 4-8 mg IV after induction unless given in truncal blocks.
- **Ondansetron**: 4 mg IV given prior to emergence
- **Haloperidol**: 1 mg IV given during skin closure
  - Reduce to 0.5mg IV in elderly patients >65 years of age

o Current vial concentration is 5mg/mL ****BE CAREFUL WITH DOSING****

**Ventral Hernia Repair Anesthesia Guideline 7.7.2021 9**

###### Pulmonary

- - - **Goals:**
- Do not give in patients with known QT prolongation (e.g. QTc>440ms)
- **Scopolamine**: No routine pre-operative scopolamine for PONV prevention
  - PUL-01- https://spec.mpog.org/Spec/Public/4
    - Median tidal volume < 10 ml/ kg predicted body weight
  - PUL-02- https://spec.mpog.org/Spec/Public/25
    - Median tidal volume ≤ 8 ml/ kg predicted body weight
  - PUL-03- https://spec.mpog.org/Spec/Public/30
    - Median PEEP ≥ 2 cm H2O (Assuming values less than 2 cm H2O is equivalent to no PEEP administered)

###### Temperature

- - **Goals**
    - TEMP-01- https://spec.mpog.org/Spec/Public/19
      - Documentation of an active warming device applied (forced air warmers, increasing room temp, and fluid warmers should be used)**.**
    - TEMP-02- https://spec.mpog.org/Spec/Public/20
      - At least one core temperature documented between Anesthesia Start and Patient out of Room.
    - TEMP-03- https://spec.mpog.org/Spec/Public/21
      - At least one body temperature measurement equal to or greater than 36 degrees Celsius (or 96.8 degrees Fahrenheit) achieved within the 30 minutes immediately before or the 15 minutes immediately after anesthesia end time.

###### Transfer of Care

- - **Goals:**
    - TOC-01- https://spec.mpog.org/Spec/Public/29: Intra-operative handoff
    - TOC-02- https://spec.mpog.org/Spec/Public/22**:** In-room to PACU handoff
    - TOC-03- https://spec.mpog.org/Spec/Public/26: OR to SICU handoff

###### Transfusion

- - **Goals:**
    - TRAN-01- https://spec.mpog.org/Spec/Public/9
      - Draw hemoglobin and/or hematocrit prior to blood transfusion.
    - TRAN-02- https://spec.mpog.org/Spec/Public/10
      - If patient received blood transfusion, Hematocrit ≤ 30% and/or hemoglobin value ≤ 10 g/dL.

**10 Ventral Hernia Repair Anesthesia Guideline**

## Postoperative Interventions

**ERAS Specific Postoperative Orders to be written *by the Surgical Service***

###### Activity Orders

- - Patient should be encouraged to be out of bed to chair within 6 hours of arrival to post-op ward.
  - Patient should be *ambulating QID*.

###### Fluid Management (by primary surgical team)

- - Review fluid orders to ensure IVF ≤ 50 mL/hr
    - IVF to total 50mL/hr (inclusive of therapeutic medication/infusions like lidocaine/ketamine) on POD 1
- **Early Refeeding based on I-FEED Paradigm** see below)[^39,^](#_bookmark32) [^40^](#_bookmark33)
  - Oral intake should be encouraged starting within 4 hours of surgery, unless contraindicated
  - Including Boost Breeze clear protein while on clear liquid diet
  - Including Impact AR protein supplement when diet advanced

1. FEED score documented once per nursing shift
   - Advance diet on POD 1 if I-FEED score 0-2 (includes protein shakes)
     - Encourage gum chewing and ensure ambulation at least TID starting POD1

■


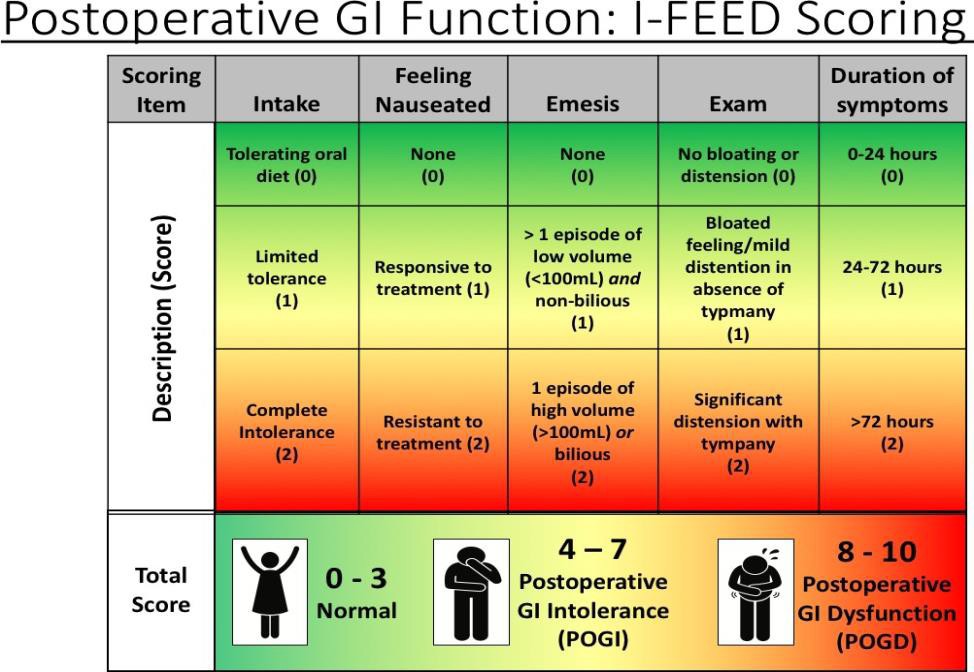

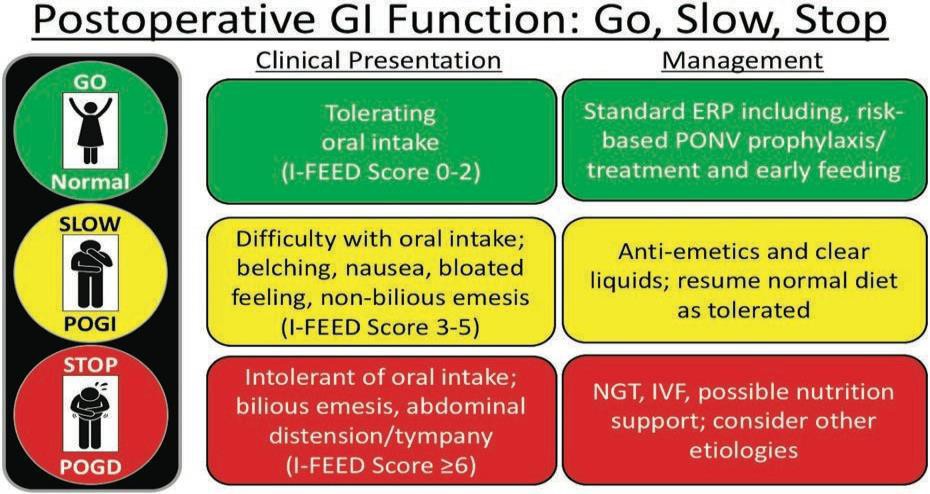


###### I-COUGH: pulmonary complication reduction measures[^41^](#_bookmark34)

- - **I** *Incentive Spirometry*: encourage patients to perform 10 times each hour.
  - **C** *Cough and Deep Breathe*: encourage patients to cough and deep breathe
  - **O** *Oral Care*: oral care BID
  - **U** *Understanding Patient Education*: educate patients about the importance of pain control and participation in activity, such as incentive spirometry and walking
  - **G** *Get out of Bed*: walking at least QID
  - **H** *Head of Bed Elevated*: head of the bed elevated between 30-45 degrees.

**Ventral Hernia Repair Anesthesia Guideline 7.7.2021 11**

**ERAS Specific Postoperative Orders to be written *by the Periop Team***

###### Postoperative Pain:

- **Thoracic Epidural:** For complex surgical cases (Rives Stoppa/Myocutaneous flap; large ventral hernias), those with pulmonary compromise, or opioid dependent patients:
  - Bupivacaine 0.1-0.15% with hydromorphone 10mcg/mL
    - Adjustment to rate and concentration determined by clinical outcomes in pain coverage, hemodynamics - discuss any adjustments with Anesthesiology Attending
    - If Ropivicaine used- start with 0.1% ropivicaine (opioid concentration does not need to change)
  - Re-evaluate each day for necessity of TEC, goal for removal by post operative day (POD) 2, prior to discharge home POD 3
    - Remove once patient having active flatus or stool, whichever earlier
    - Aim to remove TEC day prior to anticipated discharge to ensure adequate pain control on oral regimen

##### Orders to be written by Anesthesia Perioperative Consult Service * for complex cases, unless consulted otherwise[^42^](#_bookmark35)

- **Lidocaine Infusion** for 24 hours[^21,^](#_bookmark19) [^22^](#_bookmark20)
  - Continued from PACU **or after TEC removed**
  - Order for PACU to be written by Periop Service for PACU/postop admission phase of care:
    - 1 mg/min IV if <70 kg ~ 15 cc/hr
    - 1.5 mg/min IV if 70-100 kg, ~ 20 cc/hr
    - 2 mg/min IV >100 kg, ~ 30 cc/hr
  - Contraindications: Unstable heart disease, Recent MI, Heart block, Heart Failure, Electrolyte disturbances, Liver disease, Seizure disorder, Current antiarrhythmic therapy [e.g. amiodarone, sotalol] [^23^](#_bookmark21)
  - Can be extended beyond 24 hours if needed based on pain control and PO intake
  - Add lidocaine patch after infusion for localized pain[^43,^](#_bookmark36) [^44^](#_bookmark37)
- **Ketamine Infusion** for 48 hours[^18,^](#_bookmark16) [^19^](#_bookmark17)
  - Continued from PACU
  - Order for PACU to be written by Periop Service for PACU/pstop admission phase of care:
- 2.5mcg/kg/minute based on current weight
- Contraindications: glaucoma, increased intracranial pressure, severe Post Traumatic Stress Disorder, liver disease, psychosis disorder
- Can be extended beyond 48 hours if needed based on pain control and PO intake

###### Acetaminophen[^9^](#_bookmark7)

- - 1000 mg PO Q8hr starting POD0 until discharge (max 3000 mg in 24hrs)
- Then 500-1000mg PO Q8h x 3 days and then PRN.
  - Reduce to 650 mg PO Q6h if <70kg
  - Reduce to 500 mg q 8H for liver disease
- Don’t use if Child Class C liver disease

###### Gabapentin[^45-48^](#_bookmark38)

- - 300-600 mg PO q8h starting POD0 until discharge
  - Use lower dose for >65y or if patient having significant sedation/dizziness
  - Gabapentin is cleared renally and dose/frequency should be adjusted based on renal function[^49^](#_bookmark39)

**12 Ventral Hernia Repair Anesthesia Guideline**

- CrCl > 60ml/min: 300-1200mg TID
- CrCl>30-59 ml/min: 200-700mg BID
- CrCl >15-29ml/min 200-700mg once daily
- CrCl < 15- reduce dose in proportion to CrCl ~100-300 mg once daily
- ESRD requiring hemodialysis- dose based on CrCl, plus single supplemental dose of 125-250mg after dialysis
  - Up titrate dose based on sedation/tolerance if utilizing opioids

o **NSAIDs:** [**9,**](#_bookmark7) [**12,**](#_bookmark10) **50-54**

- - **Ketorolac:** 30 mg IV Q6h x 3 days
- Reduce to 15 mg IV Q6h in patients >65y, Creat <1.5, or weight <50kg
  - **Celecoxib**: 200 mg BID (alternate option)
- In patients >65y, CrCl<30, or weight <50kg - go to 100mg BID

###### Muscle Relaxants

- - Start with low dose and monitor for sedation, dizziness
  - Stagger dosing with other sedating medications
  - First line: tizanidine (Zanaflex) 2mg PO q8 hours (hold for SBP less than 110)
- Reduce dose to 1mg PO q8 hours PRN for >65 yo patients
  - Other Options:
- methocarbamol 500 q 8H (can increase to 1000mg q 8H)
  - available IV, but expensive
  - intravenous and oral dosage equivalent

###### Opioid PRN

- - Consider lowest possible dose and frequency for pain control
  - Options for opioid naive:
- **First line: Oxycodone** 5mg PO Q4 PRN pain

o Consider Hydromorphone IV PRN bolus for breakthrough pain (only write if pain not controlled with other modalities)

- - For patients on chronic opioid therapy, ensure meeting 100-125% of home oral dose, with goal of avoiding significant opioid escalation (Perioperative Service should be involved).

###### Postoperative Nausea and Vomiting (PONV)

- - **Ondansetron:** 4mg IV/PO Q6hr PRN (write:1^st^ line for nausea and vomiting)
  - **Haloperidol:** 0.5 – 1 mg IV PRN q 4-6 H PRN (write: 2^nd^ line for nausea and vomiting after Zofran)

###### Add Scopolamine patch (3^rd^ line option only if active PONV despite above)

- - - Consider scopolamine patch preoperatively if 4 or more risk factors for PONV as above.
      - Don’t use if >65y and have concern for over-sedation (ex. OSA, dementia) or anti- cholinergic use
      - Due to postoperative issues with blurry vision leading to prolonged LOS, scopolamine patch is only to be used in patients with a history of PONV or a history of motion sickness.
      - Use only one time dosing, not every 3 days unless persistent PONV
  - **4^th^ line option - Promethazine**: 6.25-12.5 mg IV/PO q 4 H PRN
    - Ensure patent IV prior to promethazine injection as extravasation can cause serious tissue injury

**Ventral Hernia Repair Anesthesia Guideline 7.7.2021 13**

- - **Consider Re-Consulting Perioperative Service by phone or to evaluate if patient’s nausea is intolerable or preventing functional recovery, to consider addition of the following adjuncts:**
    - Alternate options to consider
      - dronabinol (Marinol)- Avoid in patients > 65 yrs
      - dexamethasone (Decadron)– discuss use with surgical team before ordering
      - aprepitant (Emend) - pharmacy must be contacted for special consideration due to cost

###### For patients with pain not controlled with baseline analgesia regimen:


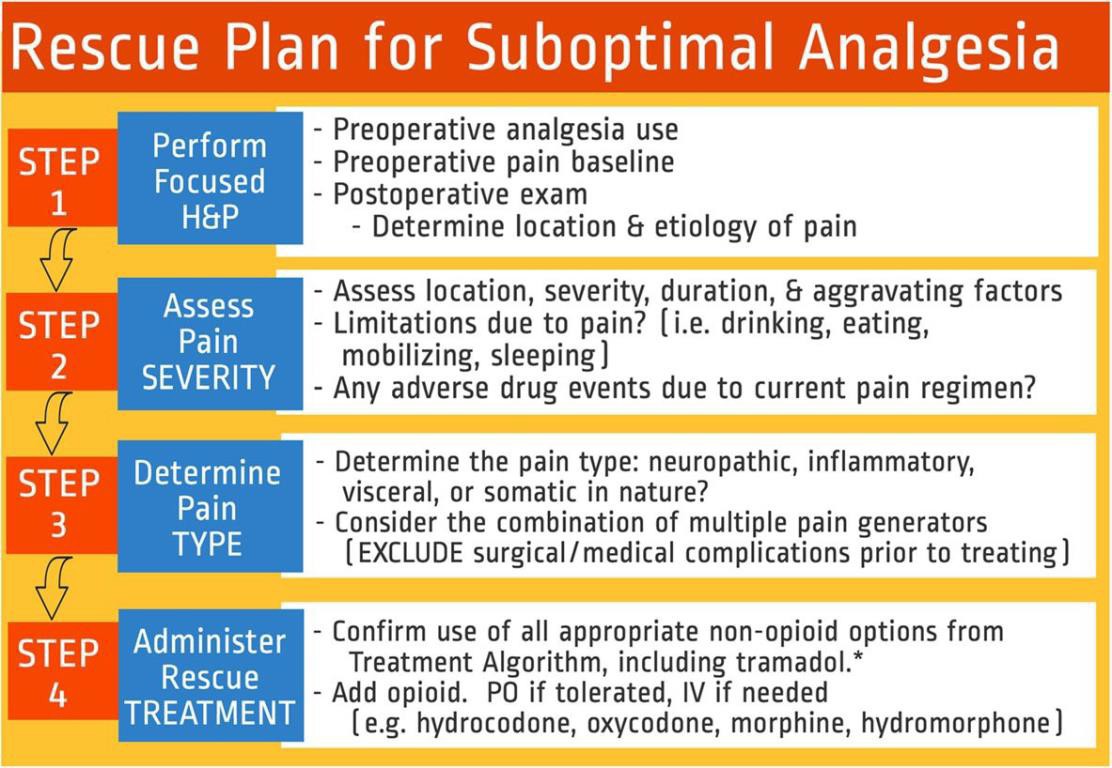


- **Consider Re-Consulting Perioperative Service by phone or to evaluate patient if patient’s pain is intolerable or preventing functional recovery, to consider additional adjuncts.**

###### Post-discharge Medications: Pain Control

***Guiding principle: Opioids should be tapered first, then multimodal medications***

###### Opioids

- - - Adhere to TN Together Opioid Prescribing Guidelines as guided in E-Star

###### Typically would recommend prescribing the opioid medication patient has been utilizing at hospital.

- - - - **Example: Oxycodone** 5mg PO Q6H PRN pain

**14 Ventral Hernia Repair Anesthesia Guideline**

- - - Taper to ~ 30-50% dose reduction by 3 days post discharge, then reduced dose q 6 H PRN x 2-3 days, then off

###### Duration of prescription:

- - - - Address duration and frequency of opioids in discharge recommendations *based on patient’s opioid utilization 24 hrs prior to discharge*.

###### If patient has not used opioids in 24 hrs prior to discharge - consider whether patient needs any opioid, or discharge with only a rescue pack of 3 pills

- - **NSAID:**
    - ibuprofen or celecoxib for 5-7 days
    - Not for inflammatory bowel disease
  - **APAP:** max of 3 grams/day for 3 days

###### Gabapentin:

- - - continue gabapentin x 14 days, or throughout opioid taper if prolonged opioid required
      - No gabapentin taper unless patient on > 300 mg q8H

o Taper example: 600mg q8h x 7 days, then 300 mg q8H x 7 days

###### Other Medications:

- - - **Muscle relaxants**
      - Continue inpatient regimen as PRN x 7 days post op or until off opioids, as long as tolerating without sedation

###### Namenda:

- - - - Continue inpatient regimen x 7 days

###### Post-discharge Medications: nausea prevention

- - Ondansetron 4 mg ODT q 6 H PRN
  - Promethazine PO 12.5 mg q 4 H PRN
  - Scopolamine patch if nausea persisting
  - Dronabinol 5 mg daily if nausea persisting

**Ventral Hernia Repair Anesthesia Guideline 7.7.2021 15**

##### References/Evidence

1. Colvin, J.; Rosen, M.; Prabhu, A.; Rosenblatt, S.; Petro, C.; Zolin, S.; Krpata, D., Enhanced recovery after surgery pathway for patients undergoing abdominal wall reconstruction. *Surgery* **2019,** *166* (5), 849- 853.
2. Orenstein, S. B.; Martindale, R. G., Enhanced Recovery Pathway for Complex Abdominal Wall Reconstruction. *Plastic and reconstructive surgery* **2018,** *142* (3 Suppl), 133S-141S.
3. Peterman, D. E.; Knoedler, B. P.; Ewing, J. A.; Carbonell, A. M.; Cobb, W. S.; Warren, J. A., Implementation of an Evidence-Based Protocol Significantly Reduces Opioid Prescribing After Ventral Hernia Repair. *The American surgeon* **2020,** *86* (11), 1602-1606.
4. Jensen, K. K., Recovery after abdominal wall reconstruction. *Dan Med J* **2017,** *64* (3).
5. Jensen, K. K.; Brondum, T. L.; Harling, H.; Kehlet, H.; Jorgensen, L. N., Enhanced recovery after giant ventral hernia repair. *Hernia* **2016,** *20* (2), 249-56.
6. Khansa, I.; Koogler, A.; Richards, J.; Bryant, R.; Janis, J. E., Pain Management in Abdominal Wall Reconstruction. *Plastic and reconstructive surgery. Global open* **2017,** *5* (6), e1400.
7. Majumder, A.; Fayezizadeh, M.; Neupane, R.; Elliott, H. L.; Novitsky, Y. W., Benefits of Multimodal Enhanced Recovery Pathway in Patients Undergoing Open Ventral Hernia Repair. *Journal of the American College of Surgeons* **2016,** *222* (6), 1106-15.
8. Warren, J. A.; Stoddard, C.; Hunter, A. L.; Horton, A. J.; Atwood, C.; Ewing, J. A.; Pusker, S.; Cancellaro, V. A.; Walker, K. B.; Cobb, W. S.; Carbonell, A. M.; Morgan, R. R., Effect of Multimodal Analgesia on Opioid Use After Open Ventral Hernia Repair. *Journal of gastrointestinal surgery : official journal of the Society for Surgery of the Alimentary Tract* **2017,** *21* (10), 1692-1699.
9. Ong, C. K.; Seymour, R. A.; Lirk, P.; Merry, A. F., Combining paracetamol (acetaminophen) with nonsteroidal antiinflammatory drugs: a qualitative systematic review of analgesic efficacy for acute postoperative pain. *Anesthesia and analgesia* **2010,** *110* (4), 1170-9.
10. Schmidt, P. C.; Ruchelli, G.; Mackey, S. C.; Carroll, I. R., Perioperative gabapentinoids: choice of agent, dose, timing, and effects on chronic postsurgical pain. *Anesthesiology* **2013,** *119* (5), 1215-21.
11. Straube, S.; Derry, S.; Moore, R. A.; Wiffen, P. J.; McQuay, H. J., Single dose oral gabapentin for established acute postoperative pain in adults. *The Cochrane database of systematic reviews* **2010,** (5), Cd008183.
12. Derry, S.; Moore, R. A., Single dose oral celecoxib for acute postoperative pain in adults. *Cochrane Database Syst Rev* **2013,** (10), CD004233.
13. Warren, J. A.; Carbonell, A. M.; Jones, L. K.; McGuire, A.; Hand, W. R.; Cancellaro, V. A.; Ewing, J. A.; Cobb, W. S., Length of Stay and Opioid Dose Requirement with Transversus Abdominis Plane Block vs Epidural Analgesia for Ventral Hernia Repair. *Journal of the American College of Surgeons* **2019,** *228* (4), 680-686.
14. Prabhu, A. S.; Krpata, D. M.; Perez, A.; Phillips, S.; Huang, L. C.; Haskins, I. N.; Rosenblatt, S.; Poulose, B. K.; Rosen, M. J., Is It Time to Reconsider Postoperative Epidural Analgesia in Patients Undergoing Elective Ventral Hernia Repair?: An AHSQC Analysis. *Annals of surgery* **2018,** *267* (5), 971-976.
15. Zhang, C.; Xu, L.; Ma, Y. Q.; Sun, Y. X.; Li, Y. H.; Zhang, L.; Feng, C. S.; Luo, B.; Zhao, Z. L.; Guo, J. R.; Jin, Y. J.; Wu, G.; Yuan, W.; Yuan, Z. G.; Yue, Y., Bispectral index monitoring prevent awareness during total intravenous anesthesia: a prospective, randomized, double-blinded, multi-center controlled trial. *Chin Med J (Engl)* **2011,** *124* (22), 3664-9.

**16 Ventral Hernia Repair Anesthesia Guideline**

1. Miller, T. E.; Thacker, J. K.; White, W. D.; Mantyh, C.; Migaly, J.; Jin, J.; Roche, A. M.; Eisenstein,

E. L.; Edwards, R.; Anstrom, K. J.; Moon, R. E.; Gan, T. J.; Enhanced Recovery Study, G., Reduced length of hospital stay in colorectal surgery after implementation of an enhanced recovery protocol. *Anesthesia and analgesia* **2014,** *118* (5), 1052-61.

1. Dhir, R.; Singh, M. R.; Kaul, T. K.; Tewari, A.; Oberoi, R., Effect of intravenous esmolol on analgesic requirements in laparoscopic cholecystectomy. *Journal of anaesthesiology, clinical pharmacology* **2015,** *31* (3), 375-9.
2. Ekhtiari, S.; Bhandari, M., Cochrane in CORR(R): Perioperative Intravenous Ketamine for Acute Postoperative Pain in Adults. *Clinical orthopaedics and related research* **2019,** *477* (11), 2411-2417.
3. Wang, X.; Lin, C.; Lan, L.; Liu, J., Perioperative intravenous S-ketamine for acute postoperative pain in adults: A systematic review and meta-analysis. *Journal of clinical anesthesia* **2021,** *68*, 110071.
4. Weinberg, L.; Rachbuch, C.; Ting, S.; Howard, W.; Yeomans, M.; Gordon, I.; McNicol, L.; James,K.; Story, D.; Christophi, C., A randomised controlled trial of peri-operative lidocaine infusions for open radical prostatectomy. *Anaesthesia* **2016,** *71* (4), 405-10.
5. Rollins, K. E.; Javanmard-Emamghissi, H.; Scott, M. J.; Lobo, D. N., The impact of peri-operative intravenous lidocaine on postoperative outcome after elective colorectal surgery: A meta-analysis of randomised controlled trials. *European journal of anaesthesiology* **2020,** *37* (8), 659-670.
6. Weibel, S.; Jelting, Y.; Pace, N. L.; Helf, A.; Eberhart, L. H.; Hahnenkamp, K.; Hollmann, M. W.; Poepping, D. M.; Schnabel, A.; Kranke, P., Continuous intravenous perioperative lidocaine infusion for postoperative pain and recovery in adults. *The Cochrane database of systematic reviews* **2018,** *6*, CD009642.
7. McCarthy, G. C.; Megalla, S. A.; Habib, A. S., Impact of intravenous lidocaine infusion on postoperative analgesia and recovery from surgery: a systematic review of randomized controlled trials. *Drugs* **2010,** *70* (9), 1149-63.
8. Murphy, G. S.; Szokol, J. W., Intraoperative Methadone in Surgical Patients: A Review of Clinical Investigations. *Anesthesiology* **2019,** *131* (3), 678-692.
9. Komen, H.; Brunt, L. M.; Deych, E.; Blood, J.; Kharasch, E. D., Intraoperative Methadone in Same- Day Ambulatory Surgery: A Randomized, Double-Blinded, Dose-Finding Pilot Study. *Anesthesia and analgesia* **2019,** *128* (4), 802-810.
10. Kurz, A.; Sessler, D. I.; Lenhardt, R., Perioperative normothermia to reduce the incidence of surgical- wound infection and shorten hospitalization. Study of Wound Infection and Temperature Group. *N Engl J Med* **1996,** *334* (19), 1209-15.
11. Hovaguimian, F.; Lysakowski, C.; Elia, N.; Tramer, M. R., Effect of intraoperative high inspired oxygen fraction on surgical site infection, postoperative nausea and vomiting, and pulmonary function: systematic review and meta-analysis of randomized controlled trials. *Anesthesiology* **2013,** *119* (2), 303-16.
12. Fasquel, C.; Huet, O.; Ozier, Y.; Quesnel, C.; Garnier, M., Effects of intraoperative high versus low inspiratory oxygen fraction (FiO2) on patient's outcome: A systematic review of evidence from the last 20 years. *Anaesth Crit Care Pain Med* **2020,** *39* (6), 847-858.
13. Oldman, A. H.; Martin, D. S.; Feelisch, M.; Grocott, M. P. W.; Cumpstey, A. F., Effects of perioperative oxygen concentration on oxidative stress in adult surgical patients: a systematic review. *British journal of anaesthesia* **2020**.
14. Akca, O.; Kurz, A.; Fleischmann, E.; Buggy, D.; Herbst, F.; Stocchi, L.; Galandiuk, S.; Iscoe, S.; Fisher, J.; Apfel, C. C.; Sessler, D. I.; Hypercapnia Trial, I., Hypercapnia and surgical site infection: a randomized trial. *British journal of anaesthesia* **2013,** *111* (5), 759-67.

**Ventral Hernia Repair Anesthesia Guideline 7.7.2021 17**

1. Fleischmann, E.; Herbst, F.; Kugener, A.; Kabon, B.; Niedermayr, M.; Sessler, D. I.; Kurz, A., Mild hypercapnia increases subcutaneous and colonic oxygen tension in patients given 80% inspired oxygen during abdominal surgery. *Anesthesiology* **2006,** *104* (5), 944-9.
2. Gustafsson, U. O.; Scott, M. J.; Schwenk, W.; Demartines, N.; Roulin, D.; Francis, N.; McNaught, C. E.; MacFie, J.; Liberman, A. S.; Soop, M.; Hill, A.; Kennedy, R. H.; Lobo, D. N.; Fearon, K.; Ljungqvist, O.; Enhanced Recovery After Surgery, S., Guidelines for perioperative care in elective colonic surgery: Enhanced Recovery After Surgery (ERAS(R)) Society recommendations. *Clin Nutr* **2012,** *31* (6), 783-800.
3. Raghunathan, K.; Shaw, A. D.; Bagshaw, S. M., Fluids are drugs: type, dose and toxicity. *Curr Opin Crit Care* **2013,** *19* (4), 290-8.
4. Kuper, M.; Gold, S. J.; Callow, C.; Quraishi, T.; King, S.; Mulreany, A.; Bianchi, M.; Conway, D. H., Intraoperative fluid management guided by oesophageal Doppler monitoring. *BMJ (Clinical research ed.)* **2011,** *342*, d3016.
5. Krajewski, M. L.; Raghunathan, K.; Paluszkiewicz, S. M.; Schermer, C. R.; Shaw, A. D., Meta-analysis of high- versus low-chloride content in perioperative and critical care fluid resuscitation. *The British journalof surgery* **2015,** *102* (1), 24-36.
6. Futier, E.; Lefrant, J. Y.; Guinot, P. G.; Godet, T.; Lorne, E.; Cuvillon, P.; Bertran, S.; Leone, M.; Pastene, B.; Piriou, V.; Molliex, S.; Albanese, J.; Julia, J. M.; Tavernier, B.; Imhoff, E.; Bazin, J. E.; Constantin, J. M.; Pereira, B.; Jaber, S.; Group, I. S., Effect of Individualized vs Standard Blood Pressure Management Strategies on Postoperative Organ Dysfunction Among High-Risk Patients Undergoing Major Surgery: A Randomized Clinical Trial. *Jama* **2017,** *318* (14), 1346-1357.
7. Wuethrich, P. Y.; Studer, U. E.; Thalmann, G. N.; Burkhard, F. C., Intraoperative continuous norepinephrine infusion combined with restrictive deferred hydration significantly reduces the need for blood transfusion in patients undergoing open radical cystectomy: results of a prospective randomised trial. *Eur Urol* **2014,** *66* (2), 352-60.
8. Wuethrich, P. Y.; Burkhard, F. C., Improved perioperative outcome with norepinephrine and a restrictive fluid administration during open radical cystectomy and urinary diversion. *Urol Oncol* **2015,** *33* (2), 66 e21-4.
9. Hedrick, T. L.; McEvoy, M. D.; Mythen, M. M. G.; Bergamaschi, R.; Gupta, R.; Holubar, S. D.; Senagore, A. J.; Gan, T. J.; Shaw, A. D.; Thacker, J. K. M.; Miller, T. E.; Wischmeyer, P. E.; Carli, F.; Evans,

D. C.; Guilbert, S.; Kozar, R.; Pryor, A.; Thiele, R. H.; Everett, S.; Grocott, M.; Abola, R. E.; Bennett- Guerrero, E.; Kent, M. L.; Feldman, L. S.; Fiore, J. F., Jr.; Perioperative Quality Initiative, W., American Society for Enhanced Recovery and Perioperative Quality Initiative Joint Consensus Statement on Postoperative Gastrointestinal Dysfunction Within an Enhanced Recovery Pathway for Elective Colorectal Surgery. *Anesthesia and analgesia* **2018,** *126* (6), 1896-1907.

1. Alsharqawi, N.; Alhashemi, M.; Kaneva, P.; Baldini, G.; Fiore, J. F., Jr.; Feldman, L. S.; Lee, L., Validity of the I-FEED score for postoperative gastrointestinal function in patients undergoing colorectal surgery. *Surgical endoscopy* **2020,** *34* (5), 2219-2226.
2. Cassidy, M. R.; Rosenkranz, P.; McCabe, K.; Rosen, J. E.; McAneny, D., I COUGH: reducing postoperative pulmonary complications with a multidisciplinary patient care program. *JAMA surgery* **2013,** *148* (8), 740-5.
3. Dahl, J. B.; Nielsen, R. V.; Wetterslev, J.; Nikolajsen, L.; Hamunen, K.; Kontinen, V. K.; Hansen, M. S.; Kjer, J. J.; Mathiesen, O.; Scandinavian Postoperative Pain, A., Post-operative analgesic effects of paracetamol, NSAIDs, glucocorticoids, gabapentinoids and their combinations: a topical review. *Acta anaesthesiologica Scandinavica* **2014,** *58* (10), 1165-81.

**18 Ventral Hernia Repair Anesthesia Guideline**

1. Bai, Y.; Miller, T.; Tan, M.; Law, L. S.; Gan, T. J., Lidocaine patch for acute pain management: a meta-analysis of prospective controlled trials. *Current medical research and opinion* **2015,** *31* (3), 575-81.
2. Saber, A. A.; Elgamal, M. H.; Rao, A. J.; Itawi, E. A.; Martinez, R. L., Early experience with lidocaine patch for postoperative pain control after laparoscopic ventral hernia repair. *Int J Surg* **2009,** *7* (1), 36-8.
3. Verret, M.; Lauzier, F.; Zarychanski, R.; Perron, C.; Savard, X.; Pinard, A. M.; Leblanc, G.; Cossi, M. J.; Neveu, X.; Turgeon, A. F.; Canadian Perioperative Anesthesia Clinical Trials, G., Perioperative Use of Gabapentinoids for the Management of Postoperative Acute Pain: A Systematic Review and Meta-analysis. *Anesthesiology* **2020,** *133* (2), 265-279.
4. Clarke, H.; Bonin, R. P.; Orser, B. A.; Englesakis, M.; Wijeysundera, D. N.; Katz, J., The prevention of chronic postsurgical pain using gabapentin and pregabalin: a combined systematic review and meta- analysis. *Anesthesia and analgesia* **2012,** *115* (2), 428-42.
5. Kumar, A. H.; Habib, A. S., The role of gabapentinoids in acute and chronic pain after surgery. *Curr Opin Anaesthesiol* **2019,** *32* (5), 629-634.
6. Peng, P. W.; Wijeysundera, D. N.; Li, C. C., Use of gabapentin for perioperative pain control -- a meta-analysis. *Pain research & management* **2007,** *12* (2), 85-92.
7. Hellden, A.; Odar-Cederlof, I.; Nilsson, G.; Sjoviker, S.; Soderstrom, A.; Euler, M.; Ohlen, G.; Bergman, U., Renal function estimations and dose recommendations for dabigatran, gabapentin and valaciclovir: a data simulation study focused on the elderly. *BMJ Open* **2013,** *3* (4).
8. Gaskell, H.; Derry, S.; Wiffen, P. J.; Moore, R. A., Single dose oral ketoprofen or dexketoprofen for acute postoperative pain in adults. *Cochrane Database Syst Rev* **2017,** *5*, CD007355.
9. Derry, S.; Cooper, T. E.; Phillips, T., Single fixed-dose oral dexketoprofen plus tramadol for acute postoperative pain in adults. *The Cochrane database of systematic reviews* **2016,** *9*, CD012232.
10. Collaborative, S. T., Safety of Nonsteroidal Anti-inflammatory Drugs in Major Gastrointestinal Surgery: A Prospective, Multicenter Cohort Study. *World J Surg* **2017,** *41* (1), 47-55.
11. Gobble, R. M.; Hoang, H. L. T.; Kachniarz, B.; Orgill, D. P., Ketorolac does not increase perioperative bleeding: a meta-analysis of randomized controlled trials. *Plastic and reconstructive surgery* **2014,** *133* (3), 741-755.
12. Walker, N. J.; Jones, V. M.; Kratky, L.; Chen, H.; Runyan, C. M., Hematoma Risks of Nonsteroidal Anti-inflammatory Drugs Used in Plastic Surgery Procedures: A Systematic Review and Meta-analysis. *Ann Plast Surg* **2019,** *82* (6S Suppl 5), S437-S445.

**Table S1: PRECIS-2 scores for trial domains**

|  | Domain | Score* | Rationale |
| --- | --- | --- | --- |
| 1 | Eligibility Criteria | 4 | This study enrolled all eligible major abdominal surgery patients within existing enhanced recovery pathways (colorectal, surgical oncology, & ventral hernia procedures) at a single academic medical center, with few exclusions |
| 2 | Recruitment Path | 4 | Ketamine infusions are an integral part of usual ERAS care at this institution, and the study was determined to pose no greater than minimal risk. This allowed for modifications to the informed consent process, developed in collaboration with the IRB. The approved process included a streamlined, one-page consent form obtained by clinical anesthesia providers at the time of anesthesia consent that required only the participant’s signature, reducing disruption to clinical workflow while maintaining ethical standards. |
| 3 | Setting | 3 | Care occurred at a single, tertiary, academic medical center |
| 4 | Organization intervention | 5 | The trial was designed to function within existing clinical workflows without dedicated research staff. Clinical providers followed their standard duties in providing enhanced recovery pathway care. In lieu of administering ketamine per the ERAS protocol, providers administered a blinded study medication (either ketamine or saline placebo). All other elements of the ERAS protocol remained consistent with usual care. |
| 5 | Flex of experimental intervention – Delivery | 4 | An OR pharmacist served on the study team and facilitated blinding and management of the study medication through routine OR pharmacy operations, eliminating the need for a separate investigational pharmacy team. |
| 6 | Flex of experimental intervention – Adherence | 4 | The study pharmacist reviewed weekly reports of study drug administration to identify cross over events. In congruence with routine practice at this institution, adherence to the abdominal ERAS protocol in this trial was monitored by an electronic, clinical dashboard that reported data to the perioperative medicine team in real-time. As with every analgesic medication in the ERAS bundle, patients reporting mild but tolerable side effects (e.g. blurry vision) with the study drug were counseled on the intended purpose and possible benefits of ketamine in their analgesic regimen. Patients were given the option to continue or cease the medication based upon their preference. Early discontinuation of a study drug order required providers to indicate a reason for cessation, which was captured in the EHR (e.g. side effects, early discharge anticipated) |
| 7 | Follow up | 5 | Study outcomes were limited to in-hospital variables and those readily derived from the EHR, with no additional data collection or patient follow-ups by research staff. Study outcomes were derived through retrospective query of the electronic health record (Epic, eStar, Verona, WI) databases using structured query language. |
| 8 | Outcome | 5 | The ultimate goal of an ERAS program is to reduce hospital length of stay, which was the primary outcome in this trial. Expeditious recovery is important to both patients and hospital systems. Secondary outcomes were also patient centered including adverse events, ICU transfer, and opioid usage. |
| 9 | Analysis | 4 | The full analysis set included all eligible patients who did not meet exclusion criteria, with the exception of a few patients excluded based on pre-specified post-randomization exclusions criteria – specifically those whose surgeries were canceled with same-day discharge or who were directly admitted to the ICU from the operating room while intubated. |

*Each domain is scored on a Likert scale 1 to 5 for features ranging from Very Explanatory (1) to Very Pragmatic (5).

PRECIS, PRagmatic-Explanatory Continuum Indicator Summary; ERAS, enhanced recovery after surgery; IRB, institutional review board; OR, operating room; ICU, intensive care unit

##### Table S2: Conversion factors for various opioids to morphine*

| **Oral Opioids:** | **Multiplication Factor to Oral Morphine (mg)** |
| --- | --- |
| Codeine (mg) | 0.1 |
| Hydrocodone (mg) | 1 |
| Hydromorphone (mg) | 5 |
| Meperidine (mg) | 0.1 |
| Methadone (mg) | 4 |
| Oxycodone (mg) | 1.5 |
| Oxymorphone (mg) | 3 |
| Tramadol (mg) | 0.25 |
| **Intravenous Opioids:** | **Multiplication Factor to Intravenous Morphine (mg)**** |
| Codeine (mg) | 0.08 |
| Fentanyl (mcg) | 100 |
| Hydromorphone (mg) | 5 |
| Meperidine (mg) | 0.125 |
| Methadone (mg) | 2 |
| Morphine (mg) | 1 |
| Nalbuphine (mg) | 1 |
| Oxymorphone (mg) | 10 |
| Sufentanyl (mcg) | 1,000 |

*Reference: Adams MCB, Sward KA, Perkins ML, Hurley RW. Standardizing research methods for opioid dose comparison: the NIH HEAL morphine milligram equivalent calculator. Pain. 2025 Feb 3. doi: 10.1097/j.pain.0000000000003529. Epub ahead of print. PMID: 39907478.

**Conversion from intravenous to oral morphine by a factor of 3

mg, milligram; mcg, microgram

##### Table S3: Odds of consuming more opioids by randomization arm and covariates

| **Characteristic** | **OR** | **95% CI** | **p-value** |
| --- | --- | --- | --- |
| **Age** (10 years) | 0.73 | (0.69, 0.77) | <**.001** |
| **BMI** (kg/m²) | 0.99 | (0.98, 1.01) | .302 |
| **Total comorbidities** | 1.14 | (1.05, 1.24) | **.002** |
| **Smoking status** |  |  |  |
| Never smoking  History of smoking | -  1.47 | -  (1.23, 1.77) | <**.001** |
| **Intended block type**  Epidural | - | - |  |
| Truncal | 1.06 | (0.84, 1.33) | .651 |
| **Intraoperative time** (hours) | 1.14 | (1.10, 1.19) | <**.001** |
| **Last ASA physical status** | 1.29 | (1.06, 1.57) | **.010** |
| **Opioid status**  Naive | - | - |  |
| Exposed | 6.26 | (4.31, 9.10) | <**.001** |
| **Anxiety**  No | - | - |  |
| Yes  **Depression** | 1.78 | (1.44, 2.20) | <**.001** |
| No | - | - |  |
| Yes | 1.23 | (0.99, 1.54) | .065 |
| **PTSD** |  |  |  |
| No | - | - |  |
| Yes | 1.51 | (0.92, 2.48) | .101 |
| **Arm** |  |  |  |
| Placebo  Ketamine | -  0.85 | -  (0.71, 1.01) | .061 |

OR, odds ratio; CI, confidence interval; BMI, body mass index; kg, kilogram; m, meters; ASA, American Society of Anesthesiologists; PTSD, post traumatic stress disorder

##### Table S4: Odds of experiencing an adverse event by randomization arm and covariates

**Rapid Response Activation Transfer to the ICU Ileus Requiring NG Decompression**

| **Characteristic** | OR | 95% CI | p-value | OR | 95% CI | p-value | OR | 95% CI | p-value |
| --- | --- | --- | --- | --- | --- | --- | --- | --- | --- |
| **Age (10 years)** | 0.72 | (0.61, 0.86) | **<.001** | 0.79 | (0.64, 0.98) | **.031** | 1.04 | (0.91, 1.18) | .591 |
| **Total comorbidities** | 1.53 | (1.32, 1.79) | **<.001** | 1.99 | (1.71, 2.32) | <.**001** | 1.24 | (1.10, 1.40) | **<.001** |
| **Intended block type** |  |  |  |  |  |  |  |  |  |
| Epidural | - | - |  | - | - |  | - | - |  |
| Truncal | 0.69 | (0.42, 1.14) | .149 | 0.4 | (0.23, 0.69) | .**001** | 0.78 | (0.51, 1.19) | .250 |
| **Opioid status** |  |  |  |  |  |  |  |  |  |
| Naive | - | - |  | - | - |  | - | - |  |
| Exposed | 1.75 | (0.96, 3.20) | .067 | 2.99 | (1.56, 5.72) | **<.001** | 0.72 | (0.36, 1.41) | .332 |
| **Randomization Arm** |  |  |  |  |  |  |  |  |  |
| Placebo | - | - |  | - | - |  | - | - |  |
| Ketamine | 1.51 | (0.85, 2.68) | .159 | 2.03 | (1.14, 3.63) | **.016** | 1.26 | (0.87, 1.84) | .225 |

ICU, intensive care unit; NG, nasogastric; OR, odds ratio; CI, confidence interval

##### Table S5: Odds of meeting early discharge milestones by randomization arm and covariates

| **Characteristic** | **OR** | **95% CI** | **p-value** |
| --- | --- | --- | --- |
| **Age (10 years)** | 0.99 | (0.90, 1.08) | .784 |
| **Total comorbidities** | 0.97 | (0.87, 1.08) | .582 |
| **Intended block type:** |  |  |  |
| Epidural | - | - |  |
| Truncal  **Opioid status:** | 6.74 | (4.25, 10.69) | **<.001** |
| Naive Exposed | - 0.83 | - (0.49, 1.40) | .479 |
| **Arm:** |  |  |  |
| Placebo Ketamine | - 0.68 | - (0.50, 0.93) | **.015** |

OR, odds ratio; CI, confidence interval

##### Table S6: Interactions between ketamine administration and patient or surgery-related covariates on length of stay and Opioid Exposure Subgroups

The OR values in the table reflect the effect of that variable on length of stay, based on the multivariable model that includes the interaction term specific to the variable at the top of each column.

PTSD, post traumatic stress disorder; OR, odds ratio; CI, confidence interval; BMI, body mass index; kg, kilogram; m, meter; hrs, hours; ASA, American Society of Anesthesiologists

* Sex and Surgical Type are only included as variables in the specific multivariable analyses evaluating a differential treatment effect between ketamine administration and that variable (i.e. either sex or surgical type).

##### Table S7: Primary and secondary outcomes for the per-protocol* analysis

**Outcome Ketamine**

**(N=521)**

**Placebo (N=652)**

**Overall (N=1173)**

**Length of stay (days)**

Mean (SD) 6.85 (5.26) 5.97 (3.90) 6.36 (4.57)

Median [Q1, Q3] 5.00 [4.00, 8.00] 5.00 [3.00, 7.00] 5.00 [3.00, 8.00]

**Total opioid consumption in MME**

Mean (SD) 144 (197) 142 (208) 143 (203)

Median [Q1, Q3] 82.5 [37.5, 169] 88.1 [45.0, 150] 82.5 [45.0, 158]

**Rapid response team activation**

Yes 21 (4.0%) 19 (2.9%) 40 (3.4%)

No 500 (96.0%) 633 (97.1%) 1133 (96.6%)

**ICU transfer**

Yes 23 (4.4%) 15 (2.3%) 38 (3.2%)

No 498 (95.6%) 637 (97.7%) 1135 (96.8%)

**Ileus requiring NG decompression**

Yes 39 (7.5%) 38 (5.8%) 77 (6.6%)

No 482 (92.5%) 614 (94.2%) 1096 (93.4%)

SD, standard deviation; Q1, quartile one; Q2, quartile two; MME, morphine milligram equivalents; ICU, intensive care unit, NG, nasogastric

*Excludes patients for whom the study intervention was discontinued early due to side effects Note: One patient who passed away prior to discharge is assigned the worst outcomes

##### Table S8: Post-hoc analysis of various measurements of hospitalization length and readmission rates

|  | **Ketamine** | **Placebo** | **Overall** |
| --- | --- | --- | --- |
| **Length of Stay*** | **(N=770)** | **(N=752)** | **(N=1522)** |
| Mean (SD) | 6.81 (5.23) | 6.01 (3.87) | 6.41 (4.62) |
| Median [Q1, Q3] | 5.00 [4.00, 8.00] | 5.00 [3.00, 7.25] | 5.00 [3.00, 8.00] |
| **rLOS** |  |  |  |
| Mean (SD) | 6.53 (5.67) | 5.72 (4.28) | 6.13 (5.05) |
| Median [Q1, Q3] | 4.43 [3.15, 7.42] | 4.33 [3.10, 7.28] | 4.39 [3.12, 7.35] |
| **CARLOS** |  |  |  |
| Mean (SD) | 2.33 (1.64) | 2.21 (1.39) | 2.27 (1.53) |
| Median [Q1, Q3] | 1.91 [1.30, 2.80] | 1.87 [1.28, 2.89] | 1.89 [1.29, 2.87] |
| Missing | 2 (0.3%) | 3 (0.4%) | 5 (0.3%) |
| **Readmission within 30 days of discharge**  Yes | 95 (12.3%) | 80 (10.6%) | 175 (11.5%) |
| No | 675 (87.7%) | 672 (89.4%) | 1347 (88.5%) |

N, number; SD, standard deviation; Q1, quartile one; Q2, quartile two; RLOS, resource length of stay; CARLOS, case mix index adjusted length of stay

*time interval between anesthesia start and hospital discharge

**Figure S1: The PRagmatic-Explanatory Continuum Indicator Summary 2 (PRECIS-2) wheel**

**Legend:** PRECIS-2 wheel is a graphical representation of scoring domains for pragmatic features of a trial. Each domain is scored on a Likert scale (1-5):

1. Very Explanatory

2. Rather Explanatory

3. Equally Pragmatic/Explanatory

4. Rather Pragmatic

5. Very Pragmatic


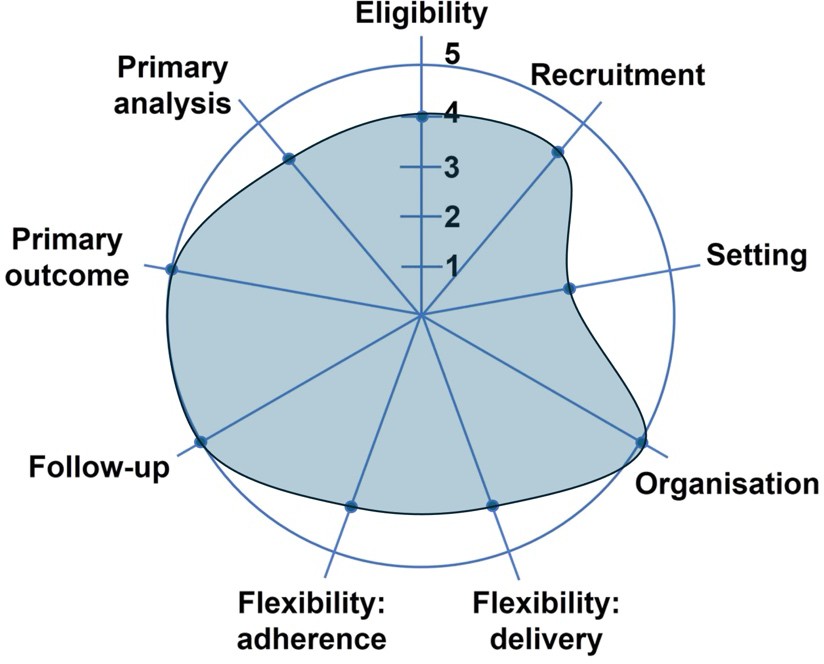


**Figure S2: Schematic illustration of the randomization for the IMPAKT ERAS trial**


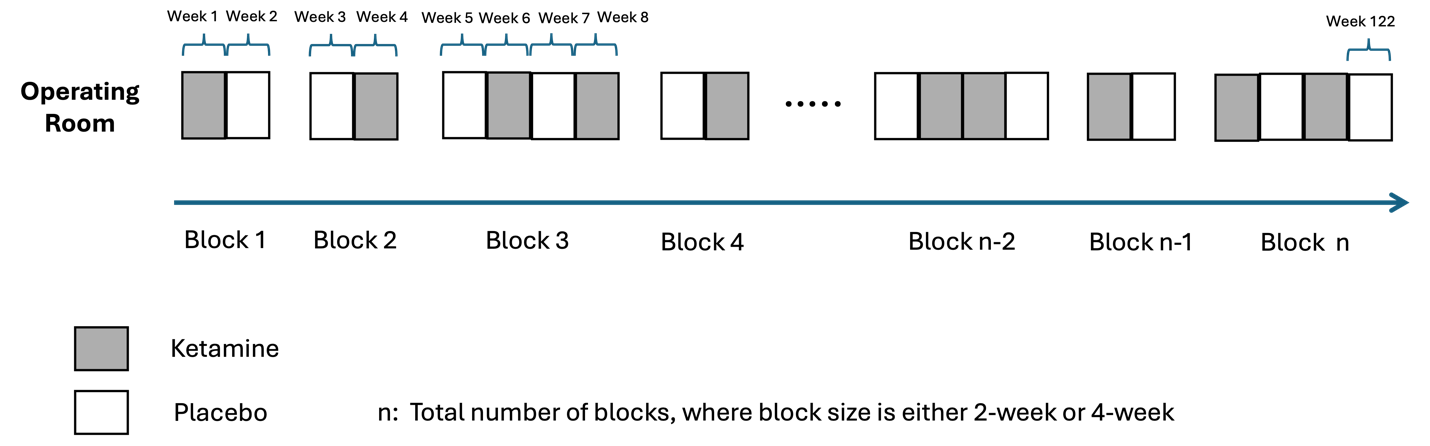

Supplement: Multimedia component 1 [file mmc1.docx]
